# Insights into individual variations in nematocyst venoms from the giant jellyfish *Nemopilema nomurai* in the Yellow Sea

**DOI:** 10.1038/s41598-019-40109-4

**Published:** 2019-03-04

**Authors:** Yang Yue, Huahua Yu, Rongfeng Li, Song Liu, Ronge Xing, Pengcheng Li

**Affiliations:** 1 Key Laboratory of Experimental Marine Biology, Institute of Oceanology, Chinese Academy of Sciences, 7 Nanhai Road, Qingdao, 266071 China; 2 Laboratory of Marine Drugs and Biological Products, Qingdao National Laboratory for Marine Science and Technology, Qingdao, 266237 China; 3 Center for Ocean Mega-Science, Chinese Academy of Sciences, 7 Nanhai Road, Qingdao, 266071 China

## Abstract

The giant jellyfish, *Nemopilema nomurai*, is widely distributed from the Eastern China Sea to the northern part of the Yellow Sea and has resulted in numerous hospitalizations in coastal areas of China, especially in Northern China. Our previous studies have revealed sting-related proteins in the venom of the jellyfish *N*. *nomurai* by using experimental and omics-based approaches; however, the variable symptoms of patients who have been stung by *N*. *nomurai* are not fully understood. This limited knowledge led to an examination of whether intraspecific variations occur in the venom of different *N*. *nomurai*. In the present study, 13 specimens of *N*. *nomurai* were collected from the Yellow Sea, and their venom was characterized by profiling differences in biochemical properties and biological activities. SDS-PAGE analysis presented recognizable differences in the number, intensity and presence of some protein bands. Moreover, enzymatic assays revealed considerable quantitative variations in metalloproteinase activity and PLA_2_-like activity. In particular, zymography assays of proteases demonstrated the general presence of abundant metalloproteinases in jellyfish nematocyst venom; however, the catalytic activities varied greatly among some specific metalloproteinases in the 28–46 kDa or 57–83 kDa range. Hemolytic assays using sheep erythrocytes suggested a predominant variance in the toxicities of different individual jellyfish venoms, with the difference between the most hemolytic and the least hemolytic venom as large as 77-fold. The current data suggested remarkable variations in the nematocyst venoms of individual *N*. *nomurai* jellyfish. These observations will provide a new understanding of the clinical manifestations induced by *N*. *nomurai* jellyfish stings and will therefore have important implications for preventing and treating jellyfish envenomations.

## Introduction

In recent decades, venomous scyphozoans have become increasingly well known for their formidable stinging ability in Eastern Asian waters. Scyphozoan *Nemopilema nomurai* Kishinouye is the main venomous jellyfish species in China, Korea and Japan, and numerous people, including tourists and fisherman, are stung in the summer months every year^1,2^. The symptoms caused by jellyfish stings can vary from mild localized pain, itch, and redness or swelling to systemic abdominal pain, vomiting, chest tightness and dyspnea^3–5^. In some cases, the occurrence of severe symptoms can be life-threatening, and patients stung by the *N*. *nomurai* jellyfish may die from acute pulmonary edema, heart failure or renal failure within several hours of being stung. The stinging ability of the *N*. *nomurai* jellyfish originates from their nematocysts in their tentacles and the venom stored in the nematocysts is very complex. Unfortunately, the specific venom components underlying jellyfish stings have so far remained elusive. Our previous studies indicated that various enzymatic components, including metalloproteinases and phospholipase A_2_s (PLA_2_s), exist in nematocyst venom extracts^6,7^, as well as in the proteome of the *N*. *nomurai* jellyfish^8^. More recently, the enzymatic components were reported to be associated with multiple organ dysfunctions and lethality in animal models and were therefore assumed to be related to the symptoms caused by jellyfish stings^9,10^. In fact, the roles of snake venom metalloproteinases and PLA_2_s in snake envenomation have been characterized^11–14^. The differences in the envenomed symptoms induced by the same jellyfish species may depend on various conditions, such as jellyfish size, area of envenomed skin, and physiological uniqueness. However, whether this discrepancy also results from variations in venom compositions, such as in the enzymatic constituents, remains unknown for most Scyphozoan jellyfish stings.

The venom compositions of venomous animals from terrestrial or marine environments are susceptible to various factors such as ontogenetic, geographical, intra- and interspecific, or even individual changes^15–18^. The variations in venom compositions among different geographic locations or ontogenetic stages have been observed, mostly in terrestrial taxa such as snakes^19,20^. In contrast to the terrestrial taxa, evidence of the venom variations in venomous marine animals has been relatively scant. One intriguing example was from studies on the complexity and variations in venom from cone snails, which could shift between predation- and defense-evoked venoms in response to predatory or defensive stimuli^21^. A recent study on venom production dynamics in sea anemone *Nematostella vectensis* also revealed great variability in venom compositions during the developmental shift from early embryonic stages to mature individuals^22^.

As one of the medically important venomous creatures in the ocean, jellyfish are of interest to the scientific community for their possible ontogenetic and geographical variations in their fish-hunting nematocyst venom. In Australian waters, the *Carukia barnesi* jellyfish was reported to be a highly toxic cubozoan that cause *Irukandji syndrome*^23^. Previous studies have demonstrated that nematocyst venoms from mature *C*. *barnesi* were distinct from those extracted from immature individuals, indicating the occurrence of ontogenetic differences in venom composition^24^. The ontogenetic switch in venom compositions was thought to correlate with a diet shift from invertebrate to vertebrate prey. In addition to *C*. *barnesi*, another cubozoan jellyfish, *Chironex fleckeri*, was also found to have similar venom ontogeny to adapt to feeding changes at different developmental stages^25^. Moreover, significant geographical variations were demonstrated in *C*. *fleckeri* venoms, which displayed remarkable differences in pharmacological effects in animal testing^26^.

The giant Scyphozoan jellyfish, *N*. *nomurai*, widely distributed in the Sea of Japan and the coastal waters of China and Korea, has resulted in countless hospitalizations in Northern China alone. However, there is no evidence so far as to whether intraspecific variation occurs in the nematocyst venoms from different *N*. *nomurai* individuals collected from different geographic sites. Therefore, the aim of this study was to provide the first insights into the venom variability in biochemical components and biological activities among different Scyphozoan *N*. *nomurai* jellyfish individuals.

## Results

### Individual jellyfish specimens collected in the Yellow Sea

**Figure 1:**
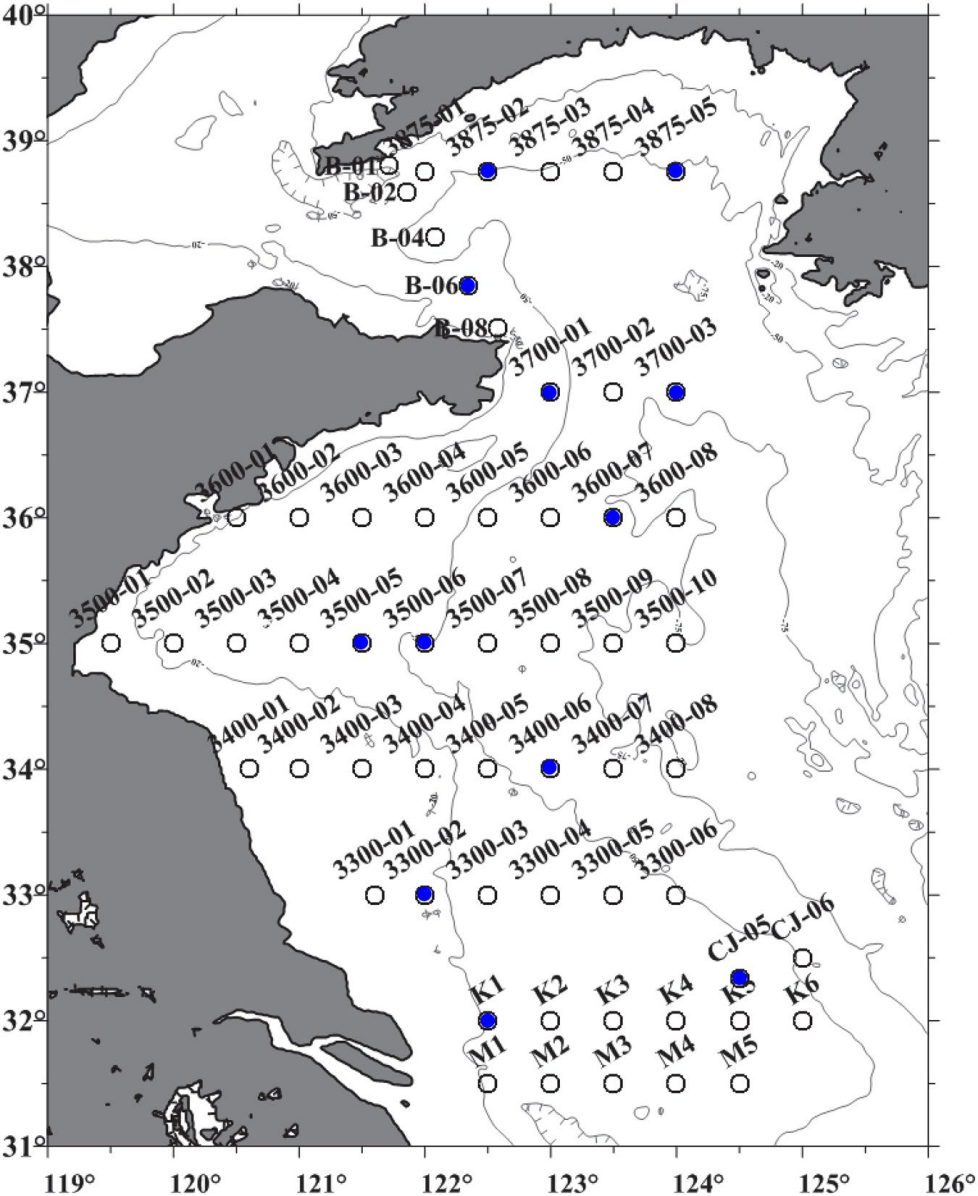
Collection stations in the Yellow Sea used by research vessel *Beidou* in the summer of 2015. Black circles with blue dots represent the 12 working stations from which 13 individual jellyfish were collected.

**Figure 2:**
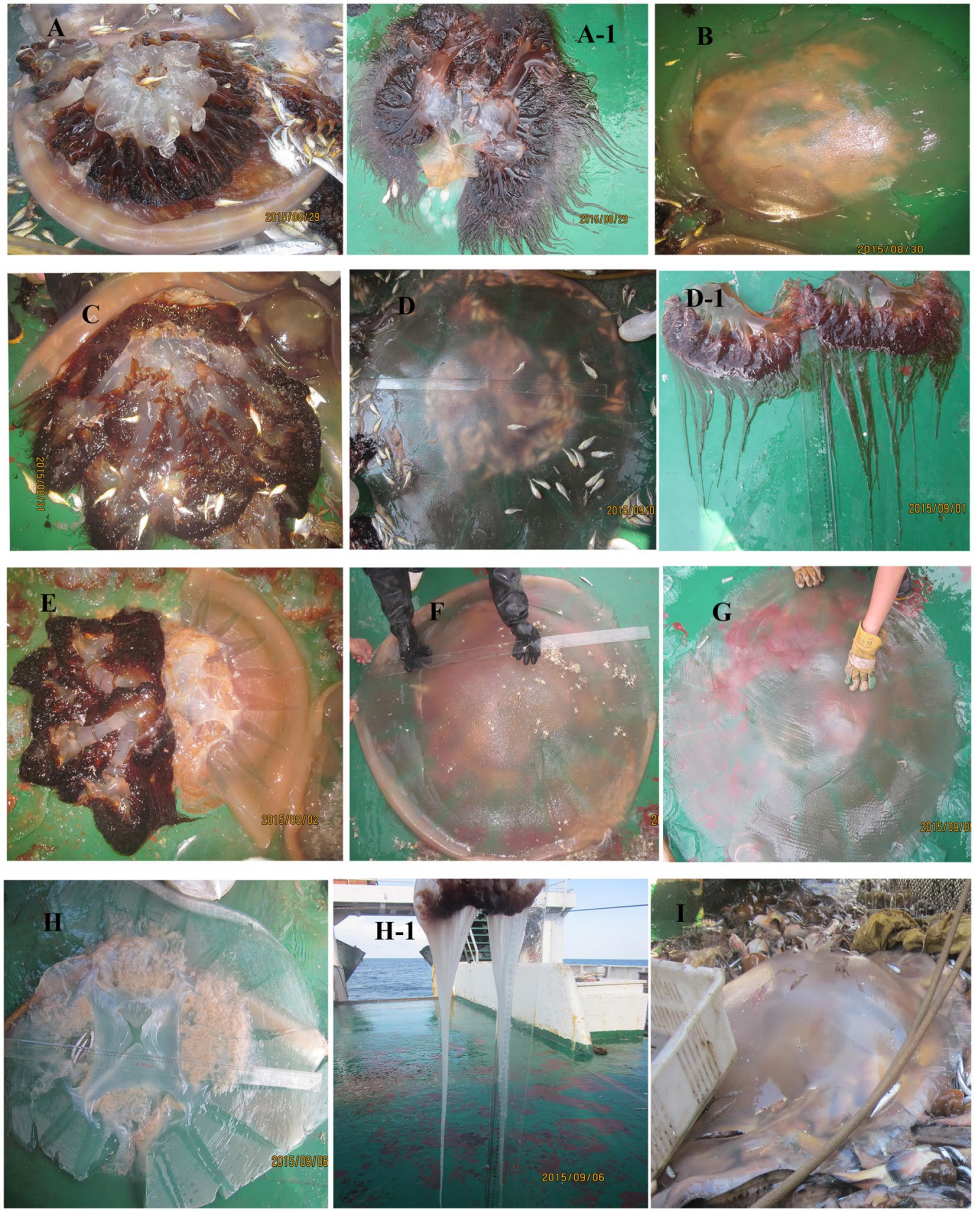
Representative photographs of individual *Nemopilema nomurai* jellyfish (**A**–**I**) and their perspective tentacle tissues (A-1, D-1, H-1). (**A**) Collected at station K1; (**B**), station CJ-05; (**C**), station 3300-02; (**D**), station 3400-06; (**E**), station 3500-06; (**F**), station 3600-07; (**G**), station 3700-03; (**H**), station B-06; (**I**), station 3875-02. All photographs were taken by the author Yang Yue.

During the summer cruises of the research vessel *Beidou* in 2015, 13 individual jellyfish specimens were captured, and their tentacles were sampled (Fig. 1, Table 1). Some representative individuals of the *N*. *nomurai* jellyfish were photographed and are presented in Fig. 2. These 13 individuals had a broad distribution across the Yellow Sea, ranging from station K1 (122°33.7570E, 32°00.4640N) to station 3875-05 (123°49.3200E, 38°44.8730N) (Fig. 2, Table 1). The *N*. *nomurai* jellyfish varied in their umbrella size from 0.9 m to 1.4 m (Table 1). Importantly, all the selected jellyfish individuals possessed long and fine tentacles, which was very supportive for obtaining an adequate number of nematocysts for venom extraction. Of note, in the northern part of the Yellow Sea, one jellyfish, J10, captured at station B06 was found to have long white tentacles (Fig. 2H), which was significantly different from the other *N*. *nomurai* jellyfish (Fig. 2A–E).

**Table 1:** Geographical distributions of 13 individual *Nemopilema nomurai* jellyfish collected in the Yellow Sea of China in 2015.

Individual Jellyfish^a^	Stations	CollectingTime (y/m/d)^b^	Longitude	Latitude	Depth(m)	Bell Size(m)
J1	K1	20150829	122°33.7570	32°00.4640	30	1.2
J2	CJ-05	20150830	124°26.6850	32°18.6870	43	1.3
J3	3300-02	20150831	122°05.6190	32°59.4119	19	1.2
J4	3400-06	20150901	122°54.4710	34°01.1410	69	1.4
J14	3400-06	20150901	122°54.4710	34°01.1410	69	1.0
J5	3500-06	20150902	122°05.8120	34°59.8460	50	1.0
J13	3500-06	20150902	122°05.8120	34°59.8460	50	0.9
J6	3500-05	20150903	121°35.1160	34°58.4920	40	1.1
J7	3600-07	20150905	123°32.6530	36°00.5600	75	1.3
J8	3700-01	20150905	123°02.7250	37°00.8830	30	1.2
J9	3700-03	20150905	123°59.2100	36°6.1980	76	1.3
J10	B-06	20150906	122°22.2720	37°48.6830	33	1.3
J11	3875-02	20150907	122°28.3420	38.45.0420	51	1.4
J12	3875-05	20150907	123°49.3200	38°44.8730	53	1.0

Notes: ^a^different individual jellyfish were tagged with a letter J plus a number. J5 was collected from 3 individual jellyfish species and their tentacles were pooled together for use. J13 had very dark colored tentacles, while the tentacles of J10 were almost white. ^b^Collection time y/m/d indicates year/month/day.

### Profiling jellyfish venom compositions by SDS-PAGE

Whether different individual jellyfish vary in their venom compositions and toxicities is of great interest. Prior to venom extraction, the most important step is to obtain sufficient and well-isolated nematocysts. The quality of the isolated nematocysts from each jellyfish individual was examined by light microscope and are showed in Fig. S1. To normalize the venom extraction process, we utilized a standard procedure to extract jellyfish venom using bead mill homogenization, as described in the Methods section. According to the procedure, approximately 0.3 grams of nematocysts was weighed and extracted with 1.5 mL of extraction buffer. The concentrations of the resulting nematocyst venoms were further quantified by Folin-Ciocalteu’s phenol reagent, and they were found to range between 0.35 and 1.88 mg/mL (Table 2).

**Table 2:** Concentrations of different jellyfish nematocyst venoms using Folin-Ciocalteu’s phenol reagent.

Jellyfish Individual Venoms	Stations	Weight of nematocysts (g)	Protein Concentration (mg/mL)
JV1	K1	0.3100	1.05
JV2	CJ-05	0.3147	0.82
JV3	3300-02	0.3156	0.35
JV4	3400-06	0.2502	0.54
JV14	3400-06	0.3069	0.60
JV5	3500-06	0.3580	0.35
JV13	3500-06	0.2621	0.50
JV6	3500-05	0.3079	0.81
JV7	3600-07	0.3038	0.78
JV8	3700-01	0.3176	1.88
JV9	3700-03	0.2516	0.65
JV10	B-06	0.3118	1.72
JV11	3875-02	0.3038	0.53
JV12	3875-05	0.3075	1.09

**Figure 3:**
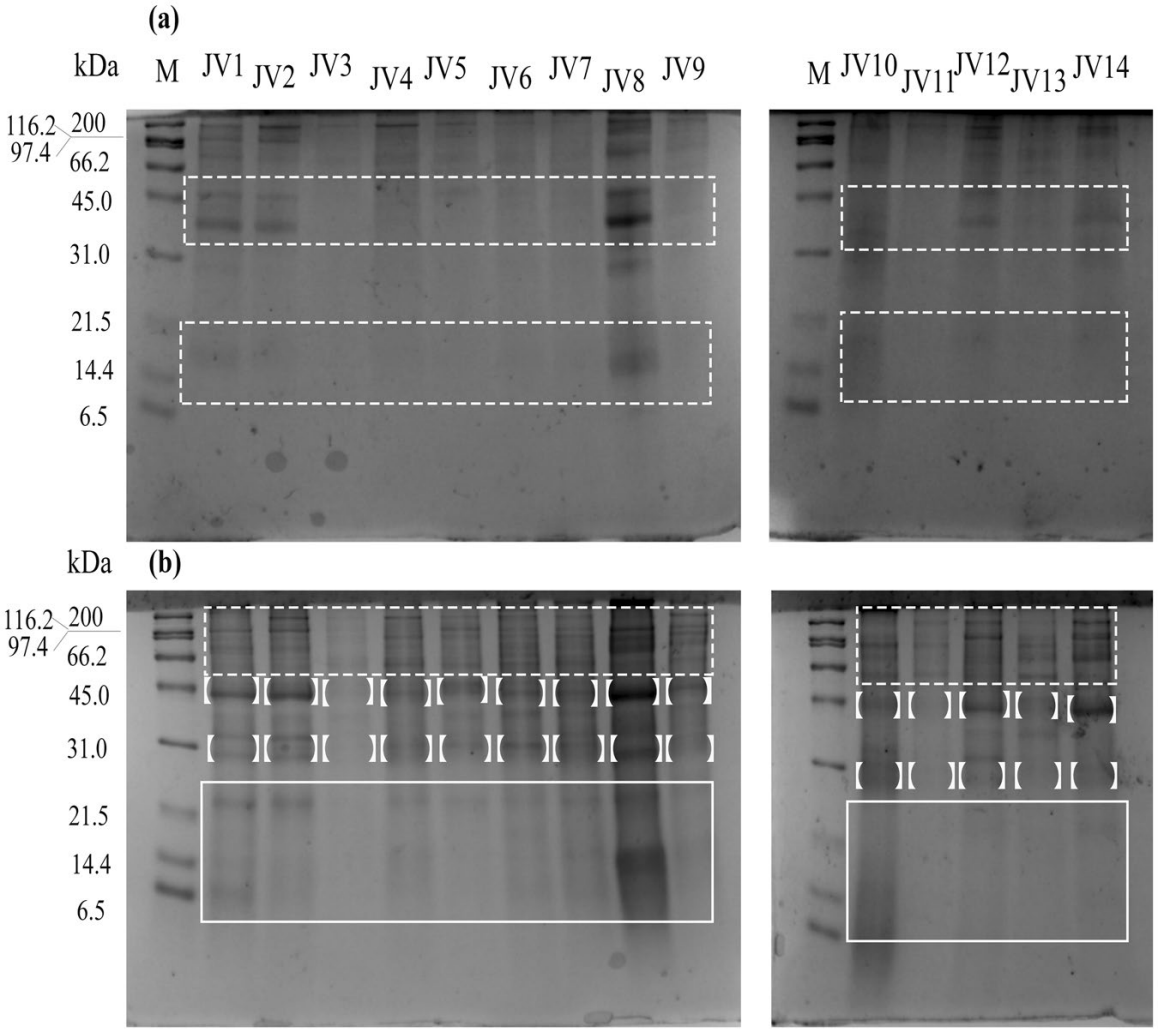
SDS-PAGE profiles of the nematocyst venoms of *Nemopilema nomurai* jellyfish. A total of 10 μg of venom proteins from individual jellyfish J1-J14 were loaded onto the gels and electrophoresis was performed at 120 V under nonreducing conditions (**a**) or reducing conditions (**b**) JV1-JV14 represent nematocyst venoms from jellyfish individuals J1-J14; M, protein markers (kDa). The differential bands were indicated by square brackets and rectangles.

The compositional variations in the 13 individual jellyfish venoms were preliminarily analyzed by SDS-PAGE under reducing and nonreducing conditions, as illustrated in Fig. 3. Obvious differences were detected in the number, intensity and presence/absence of some protein bands among the different individual jellyfish venoms. In general, the number of protein bands under nonreducing conditions was significantly less than that under reducing conditions, indicating the existence of many intermolecular disulfide bonds in jellyfish venom proteins. Under nonreducing conditions, nematocyst venoms JV1, JV2, JV8, JV12, and JV14 exhibited very similar protein band patterns, despite of the occurrence of recognizable differences in the intensity of some of the protein bands. The abundance of protein bands greatly increased under reducing conditions, and venoms from the 13 individual jellyfish were very similar, except for JV3 and JV11 in the molecular mass ranges of 22–32 kDa and 42–200 kDa. However, there were still clear noticeable variations in the presence and intensity of some venom components. For example, a protein band at 43 kDa was most intense in JV8; however, the intensity of this band was lower in JV1, JV2 and JV14. Although there is an abundant number of protein bands in the range of 43–200 kDa, careful inspections of the protein bands led to the identification of highly variable venom components with different molecular masses or intensities. Another instance of obvious venom composition variation occurred at 6–14.4 kDa under reducing conditions, in which case only JV1, JV8 and JV10 exhibited visible protein bands.

### Comparisons of enzymatic activities

**Figure 4:**
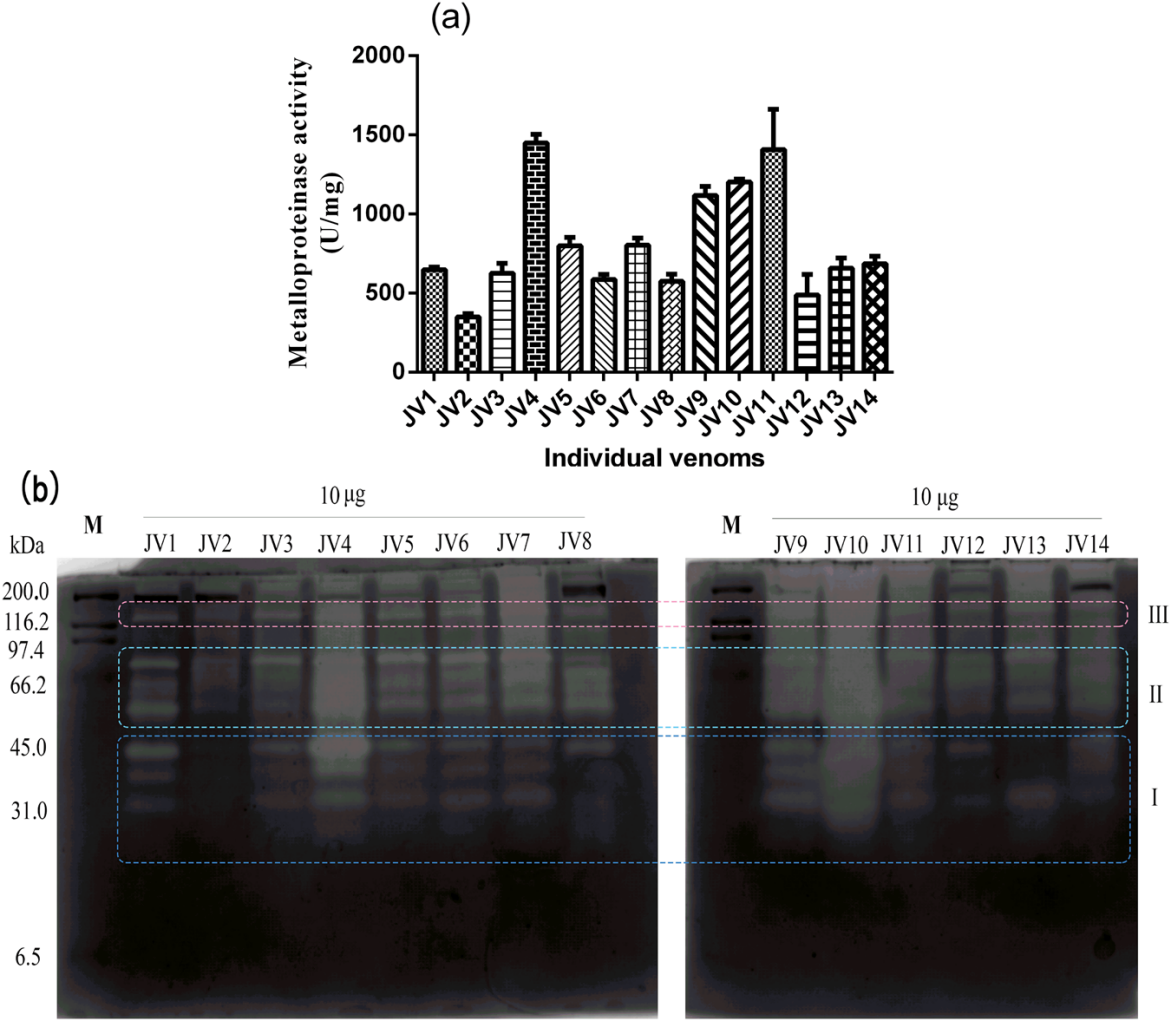
Metalloproteinase activity. (**a**) Specific activity of JV1-JV14 determined using azocasein with results expressed as U/mg from at least three replicates. (**b**) Protease zymogram of JV1-JV14. Approximately 10 μg of each venom was loaded onto the substrate gel. The presence of clear translucent bands against the blue background indicates the existence of proteolytic enzymes. The zymogram is displayed in grayscale. I, 28–46 kDa; II, 57–83 kDa; III, 139 kDa; M, marker (kDa).

The metalloproteinase activity was measured in a qualitative and quantitative manner. Specific activity was calculated to quantitatively compare the relative potency of the metalloproteinase activities of different individual jellyfish venoms. As presented in Fig. 4a, obvious variance occurred in the venoms from *N*. *nomurai* jellyfish. Of all the jellyfish individuals, J4 and J11 showed the most powerful metalloproteinase activities, with specific activities of 1449.41 ± 55.47 U/mg (n = 4) and 1408 ± 254.46 U/mg (n = 4), respectively. The metalloproteinase activity of JV9 and JV10 ranked second to JV4 and JV11. Moreover, protease zymography of the venoms were further conducted in a qualitative way using gelatin as the substrate. These results are shown in Fig. 4b. At least 9 recognizable zymolytic bands were detected in all jellyfish venoms, which included four bands at 28–46 kDa (I), four bands at 57–83 kDa (II) and one band at 139 kDa (III). The overall zymolytic band pattern shown in the zymogram was relatively stable. However, there were clear differences in the intensities and presence of some proteases among the different individual jellyfish venoms. High variance was detected among the four zymolytic bands at 28–46 kDa, indicative of venom composition variations at the intraspecies level. Most individual jellyfish venoms (JV1, JV4, JV5, JV6, JV7, JV9, JV10, JV11, JV12, and JV14) exhibited at least 3 clear zymolytic bands at 32 kDa, 39 kDa, and 46 kDa, however, venom from J8 and J13 only showed obvious degradation activity at 46 kDa and 32 kDa, respectively. Moreover, of the four zymolytic bands at 28–46 kDa, JV4 and JV10 presented the most intensive degradation activity at 46 kDa, while JV10 showed almost equivalent gelatin-degrading activity at 32–46 kDa. Similar venom composition variance was also observed among the four bands at 57–83 kDa. In addition, the total intensity of the zymolytic bands of each jellyfish venom was in accordance with the results obtained in the quantitative assay.

**Figure 5:**
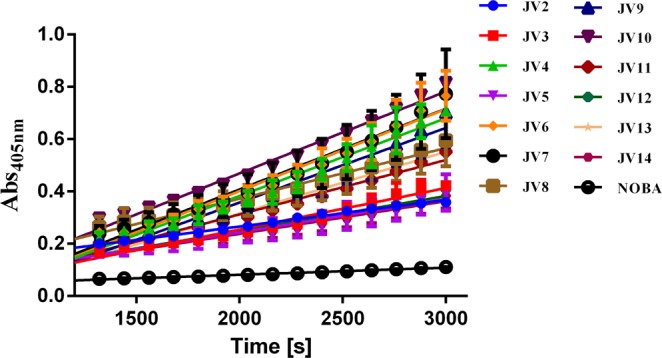
PLA_2_-like activity of different jellyfish nematocyst venoms assayed with NOBA. A total of 25 μL of JV1-JV14 was added to 200 μL of assay buffer containing 50 mM Tris-HCl, 5 mM CaCl_2_, and 100 mM NaCl, at pH 8.0 in a 96-well plate. The reactions were initiated by adding 25 μL of 1 mg/mL NOBA solution, and the absorbance was recorded for 50 min at 405 nm at 37 °C.

In addition to the metalloproteinase activity, the PLA_2_-like activity was also determined using NOBA as substrate. Figure 5 shows that venom from individual jellyfish almost linearly degraded NOBA within 20–50 min of reactions initiation. The PLA_2_-like activity was calculated as an average velocity (*v*) shown in Table 3. These results indicated that the nematocyst venoms from individual jellyfish varied significantly in their NOBA-degrading activities, and the highest PLA_2_-like activity detected in the venoms from J6 and J10 with velocity values of 7.61 ± 0.52 nM/min and 7.60 ± 0.24 nM/min, respectively. The lowest activity was found in the venom from J2 with a PLA_2_-like activity value of 2.47 ± 0.08 nM/min.

**Table 3:** NOBA degradation reaction rate calculations of different jellyfish nematocyst venoms.

Samples	R^2^	Slope (ΔAbs_405nm_/s)	reaction rate (*v*, nM/min)
NOBA	0.9800	2.713e-005 ± 5.909e-007	0.61 ± 0.01
JV1^a^	—	—	—
JV2	0.9698	0.0001018 ± 3.396e-006	2.47 ± 0.08
JV3	0.9176	0.0001583 ± 8.963e-006	3.84 ± 0.20
JV4	0.9015	0.0002969 ± 1.855e-005	7.21 ± 0.42
JV5	0.6758	0.0001273 ± 1.666e-005	3.09 ± 0.38
JV6	0.8691	0.0003137 ± 2.301e-005	7.61 ± 0.52
JV7	0.7378	0.0003084 ± 3.475e-005	7.48 ± 0.79
JV8	0.6804	0.0001937 ± 2.509e-005	4.70 ± 0.57
JV9	0.8307	0.0002851 ± 2.432e-005	6.92 ± 0.55
JV10	0.9854	0.0003130 ± 1.056e-005	7.60 ± 0.24
JV11	0.9768	0.0002079 ± 8.895e-006	5.05 ± 0.20
JV12	0.9661	0.0001334 ± 6.929e-006	3.24 ± 0.16
JV13	0.9777	0.0002358 ± 9.887e-006	5.72 ± 0.22
JV14	0.8161	0.0001249 ± 1.120e-005	3.03 ± 0.25

Note: ^a^The PLA_2_-like activity of JV1 was not obtained due to the absence of the substrate NOBA in the reaction system.

Figure S2 shows the results of hyaluronidase activity of nematocyst venoms from different individual jellyfish. Only the venom from jellyfish individual J6 exhibited a single faint zymolytic band at approximately 48 kDa. However, the zymolytic band pattern shown in this figure was differed from our previous results.

### Individual jellyfish venom variations in hemolytic activity

**Figure 6:**
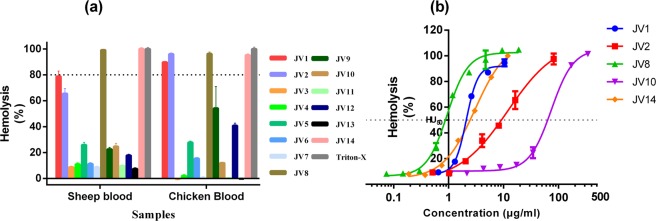
Hemolytic activity. (**a**) The hemolytic activity of JV1-JV14 against two types of blood cells, sheep erythrocytes and chicken erythrocytes at 7.0–37.6 μg/mL. A total of 50 μL of venom proteins (35.0–188.0 μg/mL in PBS) from jellyfish individuals J1-J13 was added to 200 μL of PBS-diluted erythrocytes in triplicate and then incubated at 37 °C for 30 min. The hemolytic activity was measured by examining the absorbance at 540 nm. A solution of 1% Triton X-100 in PBS and PBS alone represented 100% and 0% lysis, respectively. The hemolysis rate (%) was calculated as the percentage relative to complete lysis. (**b**) Concentration-response curves of JV1, JV2, JV8, JV10, and JV14 against 1% sheep erythrocytes. The results were expressed as the mean ± S.E.M. (n = 3). The dashed line indicates the HU_50_ values, which was defined as the concentration of protein that causes 50% lysis of sheep erythrocytes.

The hemolytic activity of nematocyst venoms from different individual jellyfish was assayed with 1% sheep or chicken erythrocytes. Prominent individual variations were found in the hemolytic activities of venoms from individual jellyfish specimens. Figure 6a presents that the nematocyst venoms from jellyfish individuals J1, J2, J8, J10 and J14 displayed potent hemolysis against two types of erythrocytes at 7.0–37.6 μg/mL concentrations. Based on these results, further experiments were performed to obtain concentration-response curves. As illustrated in Fig. 6b, the hemolytic activities of five selected jellyfish individuals exhibited significant differences. Jellyfish individual J8 was the most hemolytic with an HU_50_ value of 0.95 ± 1.04 μg/mL (n = 3), which was 77 times more hemolytic than J10 (HU_50_ = 73.82 ± 1.07 μg/mL, n = 3) (Table 4). In addition, concentration-response curves indicated similar hemolytic potency for JV1 and JV14, with HU_50_ values of 2.10 ± 1.03 μg/mL and 3.00 ± 1.04 μg/mL (Table 4), respectively.

**Table 4:** Fit results of the HU_50_ values of J1, J2, J8, J10, and J14 against sheep erythrocytes employing a four-parameter logistic curve in GraphPad Prism 6.0.

	JV1	JV2	JV8	JV10	JV14
HU_50_ (μg/mL)	2.10	11.94	0.95	73.82	3.00
Std. Error	1.03	1.18	1.04	1.07	1.04
HillSlope	4.191	0.9748	2.307	2.182	1.513
R square	0.9958	0.9850	0.9931	0.9964	0.9980

### Multivariate Analysis

**Figure 7:**
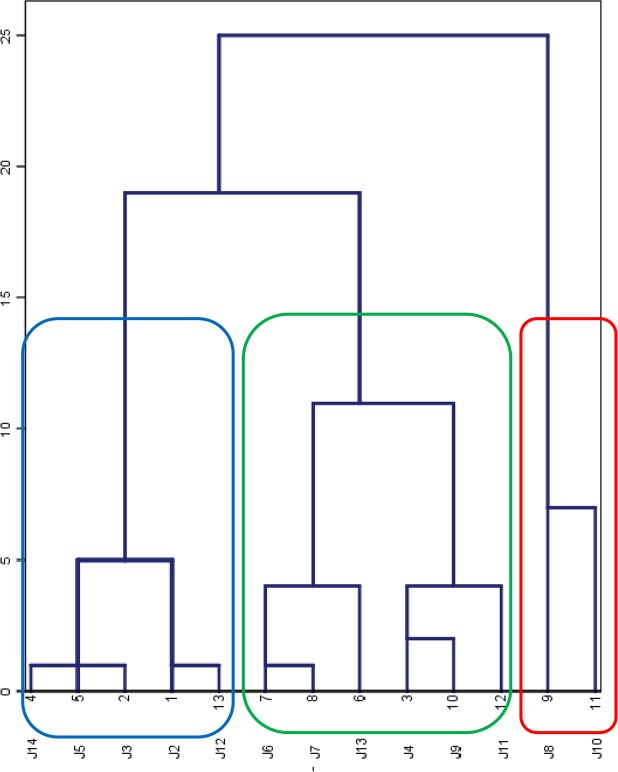
Cluster results of protein concentration and metalloproteinase and PLA_2_ activities of venom from 13 individual *N*. *nomurai* using a hierarchical clustering method. Blue, green and red outlines indicate the three groups that resulted from clustering analysis.

To provide a comprehensive view of the individual variability detected in the nematocyst venoms, a multivariate analysis was performed using a built-in clustering method in the SPSS software to estimate the variation in protein production and enzymatic activities (metalloproteinase activity, PLA_2_-like activity). Biological data from the hemolytic assays were not submitted to the multivariate analysis because not all the HU_50_ values were available in this study. The clustering results are presented in Fig. 7. Three different groups were suggested for the 13 jellyfish individuals according to the multivariate analysis. Group 1 (blue box in Fig. 7) is composed of J2, J3, J5, J12, and J14; group 2 (green box) is composed of J4, J6, J7, J9, J11, and J13; and group 3 (red box) is composed of the remaining individuals, J8 and J10. Moreover, in groups 1 and 2, the PLA_2_-like activity of the specimens appears to be the decisive factor in the formation of subgroups (Fig. 7). We found that group 3 consisted of the least number of jellyfish specimens, while the jellyfish individuals that clustered into group 3 were relatively more hemolytic than the others. Because the overall number of jellyfish specimens covered in this study (n = 13) was quite low, it was impossible to provide an accurate description of the intra-species variation that occurs in *N*. *nomurai* jellyfish. However, the clustering results offer new insight on the biochemical and biological variations that occur in jellyfish venom components.

## Discussion

In the present study, significant differences were observed through the analysis of the chemical compositions and hemolytic potencies of nematocyst venoms from 13 individual *N*. *nomurai* jellyfish, suggesting the occurrence of individual variations in Scyphozoan *N*. *nomurai* jellyfish venom. This venom variation, which occurred at the intraspecific level, may contribute to the understanding of the variability in the symptomatology that occurs in envenomed humans by the same Scyphozoan jellyfish species.

As previously mentioned, the *N*. *nomurai* jellyfish is vastly distributed across the Yellow sea, in Korean waters and in the Sea of Japan due to the currents and their swimming ability. However, it was strongly suggested that the *N*. *nomurai* population in the Sea of Japan is transported there mainly from the coastal waters along China and the western Korean Peninsula^27^. A recent life-style study pointed out that the pelagic stages of *N*. *nomurai* were principally distributed in the Yellow Sea^28^. Therefore, specimens collected in the Yellow Sea will be representative to study individual variations in venom. On the summer cruises, different morphological phenotypes of *N*. *nomurai* jellyfish, including varying tentacle color, bell size, thickness and intensity, were observed. Previously published data on the bell size in the Yellow Sea demonstrated that the bell diameters of *N*. *nomurai* jellyfish in August of 2012 and 2013 generally varied from 63 ± 21 cm to 83 ± 21 cm^28^. In this study, the *N*. *nomurai* jellyfish selected for research were relatively large individuals with bell diameters ranging from 0.9 m to 1.4 m, with most of them possessing fine tentacles (Fig. 2A–D). As we know, *N*. *nomurai* has colored tentacles, although to different extents, which are believed to be related to dinoflagellate^29^. However, one jellyfish individual, J10, captured from station B-06 possessed pure white tentacles, with an absence of any color, which is rare to observe (Fig. 2H,H-1). In the present study we did not confirmed that J10 individual belonged to *N*. *nomurai* species at the molecular level. However, its morphology and nematocyst type strongly indicate that J10 does belong to *N*. *nomurai* (Figs 2H and S1J). Moreover, the intraspecific variability in jellyfish tentacle color has been observed in another Scyphozoan *Cassiopea andromeda* in the field^29^. These observations suggest that great morphological variance occurs in *N*. *nomurai* jellyfish and that these morphological variations may have resulted from various factors, such as genetic changes, feeding ecology, and prey/defense adaptations.

Whether intraspecies venom variation occurs in the giant Scyphozoan *N*. *nomurai* has not been determined before. In the present study, profiling the biochemical compositions by reducing or nonreducing SDS-PAGE demonstrated considerable venom variation in the abundance, intensity and presence of specific proteins. Interestingly, we found two jellyfish individuals (J4, J14) collected at the same geographic site that exhibiting visible differences in the intensity of one protein band at 38 kDa under nonreducing conditions and at 43 kDa under reducing conditions (Fig. 3). Moreover, their venoms differed greatly in enzymatic and hemolytic activities (Figs 4 and 6). Considering the wide distribution of jellyfish individuals collected in this study and the low number of specimens collected in the Yellow Sea, we cannot conclude whether geographic variations also contributed to the individual venom differences of *N*. *nomurai* jellyfish. In previous studies, the geographic variation was reported to influence the venom composition of the *C*. *fleckeri* jellyfish which is distributed in western and eastern Australia^26^.

Considering that most of the protein species shown in the SDS-PAGE of jellyfish nematocyst venom were not fully characterized or identified to date, it is almost impossible to determine the chemical nature of a specific protein band that was variable among the individual jellyfish venoms. However, in our previous studies we demonstrated that *N*. *nomurai* jellyfish venom possessed significant enzymatic activities including metalloproteinase activity and PLA_2_-like activity^6,7^. Additionally, metalloproteinases and PLA_2_s from various venomous animals have strongly demonstrated their envenoming-related nature^12,30^. Moreover, a recent study has suggested that the *Cyanea capillata* jellyfish venom metalloproteinases were potentially involved in hemorrhagic injuries and necrosis in TE-induced delayed jellyfish envenomation syndrome^31^. Therefore, we chose to characterize the metalloproteinase and PLA_2_-like activities as venom componential variations occurring in *N*. *nomurai* jellyfish. Individual variations in metalloproteinase activity have been observed at inter- and intraspecies levels in many venomous animals such snakes and scorpions^32–35^. Previous reports have also revealed that the venom from various jellyfish species, including *Carybdea alata* and *Olindias sambaquiensis*, exhibited significant metalloproteinase activity^36,37^. However, whether the metalloproteinases in jellyfish venom vary at the intraspecies level has been elusive. This study demonstrated, for the first time, that individual variations in metalloproteinase activity occurred in *N*. *nomurai* jellyfish venom. Our results showed that there was considerable metalloproteinase activity in *N*. *nomurai* venom, which was in accordance with our previous study. Moreover, this study also suggested a general and conservative occurrence of various metalloproteinases in *N*. *nomurai* jellyfish venom, in view of the observation that almost all of the venoms from *N*. *nomurai* jellyfish exhibited a highly similar gelatin-degrading pattern at 28–139 kDa (I, II, III in Fig. 4). However, the enzymatic activity of some specific proteases varied greatly by gelatin zymogram. Moreover, the molecular masses of the identified proteases by gelatin zymogram were relatively high at 28–139 kDa, with no zymolytic bands found below 25 kDa. The zymolytic banding pattern of jellyfish venom was absolutely different from that observed in most snake venoms^33,38,39^. Snake venom metalloproteinases exhibit an obvious phenotypic shift from a P-III-rich to P-I-rich type profile during ontogenetic stages from juvenile to adult^40^. The shift in the metalloproteinase phenotype in snakes was assumed to correlate with diet adaptation to larger prey in adults. Ontogenetic changes in the venom of the Cubozoan *C*. *barnesi* jellyfish have also been reported for adaptive diet shifts from crustaceans for immature medusae to larval fish for mature medusae^25^. As a result, the phenomenon that the mature *N*. *nomurai* jellyfish individuals maintain an abundant but variable group of metalloproteinases was presumed to correlate to prey digestion. In particular, in some working stations, there were many larval fish found in tentacles of captured *N*. *nomurai* jellyfish (Fig. 2A–D). In addition to metalloproteinase activity, quantitative determination of the PLA_2_-like activity of the venoms from different jellyfish individuals also suggested clear differences.

To determine the variation in the toxicity of venom from *N*. *nomurai* jellyfish individuals, a cell-based hemolytic model was used to characterize the toxicity of various jellyfish venoms. Our results revealed predominant biological variations in the hemolytic activities of individual *N*. *nomurai* venoms, with a 77-fold difference between the highest hemolytic potency and the lowest hemolytic index (Fig. 6). However, the variance in toxicity may be underestimated because five out of the thirteen individual jellyfish venoms were found to exhibit sensitive concentration-response effects in hemolytic activity. Further inspection of these five individual jellyfish (J1, J2, J8, J10, J14) with potent hemolysis revealed a relatively random distribution across the Yellow Sea, and the jellyfish venom toxicity did not follow any obvious geographic pattern. Of note, venom from the white tentacles of jellyfish individual J10 exhibited considerable hemolytic activity with an HU_50_ value of 73.82 ± 1.07 μg/mL, which was higher than those from the colored tentacles of most of the other jellyfish individuals. This observation may suggest that jellyfish tentacle color is not related to toxicity.

In conclusion, we suggest, for the first time, that there are considerable individual variations in nematocyst venoms from *N*. *nomurai* jellyfish in the Yellow Sea. The venom variations were reflected not only in the biochemical characterization of venom protein profiles but also in the great variability in hemolytic activities of individual jellyfish venoms. Examination of the metalloproteinase and PLA_2_-Like activities revealed that the enzymatic components in jellyfish venom varied on intraspecies level. Of note, the combined analysis of metalloproteinase activities indicated a relatively stable group of proteases (i.e., metalloproteinases) in jellyfish nematocyst venom, but the catalytic ability of some specific proteases was variable. The variance in venom compositions may be important for elucidating the venom phenotypes of *N*. *nomurai* jellyfish in the near future. Moreover, profound differences were observed in the hemolytic activities of individual venoms from *N*. *nomurai* jellyfish. The biochemical and biological variations in Scyphozoan jellyfish venom may have important implications for understanding the varying symptoms of humans envenomed by *N*. *nomurai* jellyfish. Our results may offer valuable information on the venom variations among *N*. *nomurai* jellyfish individuals; however, our study is obviously limited by the lack of large number of specimens and the inability to identify the differential bands or specific differential compositions. Therefore, there is plenty of work to do in the future to uncover the venom compositions responsible for Scyphozoan jellyfish envenomations, and to understand the ecological roles of specific venom components, such as metalloproteinases, in the prey and defense adaptations of *N*. *nomurai* jellyfish.

## Materials and Methods

### Jellyfish collection

The jellyfish *N*. *nomurai* were collected in the Yellow Sea during the summer cruises of research vessel *Beidou* from August 26, 2015 to September 9, 2015. On the cruise, 56 stations were set to carry out trawl surveys to investigate the fishery resources. In actuality, there were 53 stations capturing different numbers of *N*. *nomurai* jellyfish. At each working station, captured jellyfish were excised to obtain their tentacles. During the collection, 13 jellyfish specimens with relatively long and complete tentacles were specially collected from 12 out of the 53 working stations (Fig. 1). Detailed collection information is listed in Table 1. After capture, the fishing tentacles of *N*. *nomurai* jellyfish were excised manually on the deck. To avoid the nematocyst discharging as much as possible, a Styrofoam box full of bags of ice was used during the tentacle collection. The excised fishing tentacles from each jellyfish individual were pooled into plastic zip-pack bags for storage at −20 °C. Once the RV *Beidou* docked, all samples were immediately transported to the laboratory and stored at −80 °C for long-term preservation.

### Venom extraction

Nematocysts were isolated from the tentacles of *N*. *nomurai* jellyfish according to our previous report^6^. Briefly, tentacles frozen at −80 °C were taken out and added to natural seawater precooled at 4 °C for autolysis. To speed up the detachment of nematocyst from the tentacle tissues, water exchanges were conducted for 4–6 daysat 24 h interval. Then, the debris were removed using a 200-mesh (74 μm) plankton net, and the resulting filtrates were further centrifuged at 1000 × g for 15 min at 4 °C. After gently discarding the supernatant, the sediments were washed with cold venom extraction buffer (VEB, 20 mM ${\rm{P}}{{\rm{O}}}_{4}^{3-}$, 150 mM NaCl, pH 7.4) several times. Finally, the nematocysts were obtained by centrifugation at 10000 × g for 15 min at 4 °C. The very inner parts of the sediments (i.e., the nematocysts) were obtained as the material for venom extraction. The resultant nematocysts were photographed employing an Axio Imager M2 (Carl Zeiss, Oberkochen, Germany). The pictures of nematocysts from 13 *N*. *nomurai* jellyfish individuals are presented in Fig. S1A–N.

Nematocyst venom was extracted with a bead mill homogenizer according to a previous method with minor modifications^41^. Briefly, approximately 0.3 g of nematocysts was weighed and then suspended by adding 1.5 mL of cold VEB in 2 mL screw top vials loaded with equiponderant glass beads. The vials were fixed onto the bead mill and shaken four times at 4600 rpm for 20 s, and then, the vials were placed on ice for 60 s at each interval. After extraction, the supernatants were pipetted off and centrifuged at 20000 × g for 15 min at 4 °C. The resulting supernatants were used as *N*. *nomurai* jellyfish venom in this study. For convenience, the venoms from different jellyfish individuals were labeled as JV1-JV14 and stored at −80 °C for use. Protein concentrations were determined using Folin-Ciocalteu’s phenol reagent (DingGuo ChangSheng Biotechnology Co. Ltd, Beijing, China) according to the manufacturer’s instructions.

### SDS-PAGE

Electrophoresis was performed according to Laemmli’s method^42^ using 12% polyacrylamide gel under reducing and nonreducing conditions. Briefly, 10 μg of venom protein from 13 jellyfish individuals or 5 μL of molecular weight standard was loaded into precast electrophoresis gels (Nanjing Jiancheng Bioengineering Institute, Nanjing, China) and separated in 1% SDS running buffer containing 25 mM Tris and 192 mM glycine in a Mini-PROTEAN Tetra apparatus (Bio-Rad, Hercules, CA, USA) under reducing and nonreducing condition. Venom samples were incubated at 100 °C for 5 min in the presence of β-mercaptoethanol under reducing conditions. Electrophoresis was carried out at 120 V for approximately 90 min at 4 °C. Gels were stained with 0.25% Coomassie Brilliant Blue R-250, and then photographed with gel imaging software and further analyzed using Quality One 4.6.2 software (Bio-Rad, Hercules, CA, USA). The Bio-Rad broad molecular standard includes the following proteins: Aprotinin, 6.5 kDa; lysozyme, 14.4 kDa; trypsin inhibitor, 21.5 kDa; carbonic anhydrase, 31.0 kDa; ovalbumin, 45.0 kDa; serum albumin, 66.2 kDa; phosphorylase b, 97.4 kDa; β-galactosidase, 116.25 kDa; and myosin, 200.0 kDa.

### Proteolytic activity

Proteolytic activities were measured using azocasein according to our previous methods^6^. Briefly, 10 μL of venom from different jellyfish individuals was added to 1.5 mL centrifuge tubes, and the reactions were initiated by adding 90 μL of substrate solution (azocasein, 5 mg/mL in 50 mM Tris-HCl, 100 mM NaCl, 5 mM CaCl_2_, pH 8.8). After incubation at 37 °C for 90 min, the reactions were terminated by adding 200 μL of 0.5 M trichloroacetic acid. The centrifuge tubes were placed at room temperature for 30 min and then centrifuged at 10000 × g for 15 min. Next, 150 μL of supernatant was transferred to a 96-well plate and an equal volume of 0.5 M NaOH was added. The absorbance was immediately monitored at 450 nm in an Infinite M100 plate reader (Tecan Group Ltd., Männedorf, Switzerland). One unit of activity was defined as an increase of 0.01 absorbance units at 450 nm, and the results were expressed as specific activity (U/mg).

### PLA_2_-like activity

PLA_2_-like activity was determined employing the PLA_2_-specific substrate 4-nitro-3-octanoyloxybenzoic acid (NOBA)^43^. Briefly, 25 μL of nematocyst venoms from 13 jellyfish individuals was added to 200 μL of assay buffer (containing 50 mM Tris-HCl, 5 mM CaCl_2_, 100 mM NaCl, pH 8.0), in a 96-well plate. The reactions were then initiated by adding 25 μL of NOBA solution (dissolved in acetonitrile, 1 mg/mL). The mixtures were subsequently incubated for 50 min at 37 °C, and the absorbance was recorded at 405 nm. The reactions were repeated twice. The reaction rate (*y*) was expressed as nmol/L (nM) of colored product per minute. The extinction coefficient of the colored products at 405 nm was determined to be 3.5318 mM^−1^ cm^−1^ according to our previous study^6^.

### Zymography of proteases

The proteolytic enzymes in jellyfish nematocyst venom were examined using zymography methods^44^. Briefly, the substrate gelatin was polymerized into 12% SDS-PAGE gels at a final concentration of 0.2% (w/v). Then 10 μg of venom proteins from jellyfish individuals J1-J14 was added to the substrate gels and electrophoresis was performed at 120 V for approximately 120 min under nonreducing conditions. To maintain the enzymatic activity for as long as possible, the electrophoresis apparatus was surrounded by ice. When the electrophoresis finished, the substrate gels were taken out and washed twice for 40 min with 2.5% Triton X-100, and then further immersed twice in assay buffer (50 mM Tris-HCl, 200 mM NaCl, 5 mM CaCl_2_, pH 8.8) for another 30 min. Next, the substrate gels were incubated with the assay buffer for 24 h and then stained with 0.25% Coomassie Brilliant Blue R-250. The enzymatic activity was visualized as a transparent zone against a blue background after destaining for 3 hours with a methanol: glacial acetic acid: distilled water (5:1:4) solution.

### Hemolytic activity

Comparisons of the hemolytic activities of individual jellyfish venoms JV1-JV14 were determined using two types of blood cells, sheep erythrocytes and chicken erythrocytes, as described previuosly^45^. Heparinized blood from sheep and chicken were purchased from Nanjing Maojie Technology Co. Ltd. (Nanjing, China). The erythrocytes were obtained from the heparinized blood by centrifugation at 3000 × g for 10 min at 4 °C. The resulting erythrocytes were washed three times in sterile phosphate-buffered saline (PBS, 20 mM ${\rm{P}}{{\rm{O}}}_{4}^{3-}$ 150 mM NaCl, pH 7.4) and centrifuged at 3000 × g for 10 min at 4 °C. The erythrocytes were further diluted in PBS and adjusted an absorbance at 540 nm of approximately 1.0 when 100% hemolysis occurred. To compare the hemolytic activity, 200 μL of PBS-diluted erythrocytes was added to 50 μL of venom proteins (35.0–188.0 μg/mL in PBS) from different jellyfish individuals in triplicate in 1.5 mL microcentrifuge tubes on ice. The samples were incubated at 37 °C for 30 min. Next, the samples were placed on ice for 5 min and centrifuged at 3000 × g for 5 min at room temperature, and then, the supernatants (200 μL) were transferred to a 96-well plate. The absorbance of released hemoglobin was measured at 540 nm. Solutions of 1% Triton X-100 in PBS and PBS alone were used as references to represent 100% and 0% lysis, respectively. Hemolysis rates were calculated as percentages relative to complete lysis. HU_50_ values, defined as the concentration of protein that causes 50% lysis of sheep erythrocytes, were determined for the venoms exhibiting powerful hemolytic potential. The hemolysis concentration-response curves were each fit with a four-parameter logistic curve in GraphPad Prism 6.0 (GraphPad software, San Diego, CA, USA).

### Multivariate Analysis

The cluster analysis was performed using the unsupervised hierarchical clustering method based on squared Euclidean distances^40^. The variables used in the analysis were protein concentration, metalloproteinase activity and PLA_2_ activity of the venoms. In this study, the 13 jellyfish individuals collected from 12 stations across the Yellow Sea varied little in bell size and most of them were at same developmental stages, so the impact of jellyfish size on the venom variations was omitted from the analysis. Moreover, considering the facts that only one or two jellyfish individuals were collected at each station, which were too short in number to represent the overall jellyfish samples of the geographical location of each station, so the geographic information was also not submitted to the multivariate analysis. Therefore, any clustering that might separate the samples would be only based on venom features. The statistical analysis was carried out using the SPSS software (version 22.0 for Windows, SPSS Inc., Chicago, IL, USA).

### Statistical analysis

The results were expressed as the mean ± S.E.M. The significance of differences between the means of various experimental groups was analyzed by an analysis of variance (ANOVA), followed by Tukey’s multiple comparisons test built-in Graphpad Prism 6.0 (GraphPad software, San Diego, CA, USA). *p < 0.05 was considered statistically significant.

## Supplementary information

Supplementary Information

## Data Availability

All data generated or analyzed during this study are included in this published article (and its Supplementary Information files).
