# Supplementary material for: Insights into individual variations in nematocyst venoms from the giant jellyfish Nemopilema nomurai in the Yellow Sea
Source: Sci Rep. 2019 Mar 4;9:3361. doi: 10.1038/s41598-019-40109-4 (PMC6399247; doi:10.1038/s41598-019-40109-4)
Supplement: Supplementary file 1 — Supplementary Information [file 41598_2019_40109_MOESM1_ESM.pdf]

Supplementary Information

Insights into individual variations in nematocyst venoms from the giant jellyfish *Nemopilema nomurai* in the Yellow Sea

Yang Yue [1,3], Huahua Yu [1,2,3,*], Rongfeng Li [1,2,3], Song Liu [1,2,3], Ronge Xing [1,2,3], Pengcheng Li [1,2,3,*]

[1]Key Laboratory of Experimental Marine Biology, Institute of Oceanology, Chinese Academy of Sciences, 7 Nanhai Road, Qingdao 266071, China;

[2] Laboratory of Marine Drugs and Biological Products, Qingdao National Laboratory for Marine Science and Technology, Qingdao, 266237, China;

[3]Center for Ocean Mega-Science, Chinese Academy of Sciences, 7 Nanhai Road, Qingdao, 266071, China

*Author to whom correspondence should be addressed; E-Mail: pcli@qdio.ac.cn (P.-C.L.); yuhuahua@qdio.ac.cn (H. -H.Y).

Photographs of nematocysts from jellyfish *Nemopilema nomurai* individuals

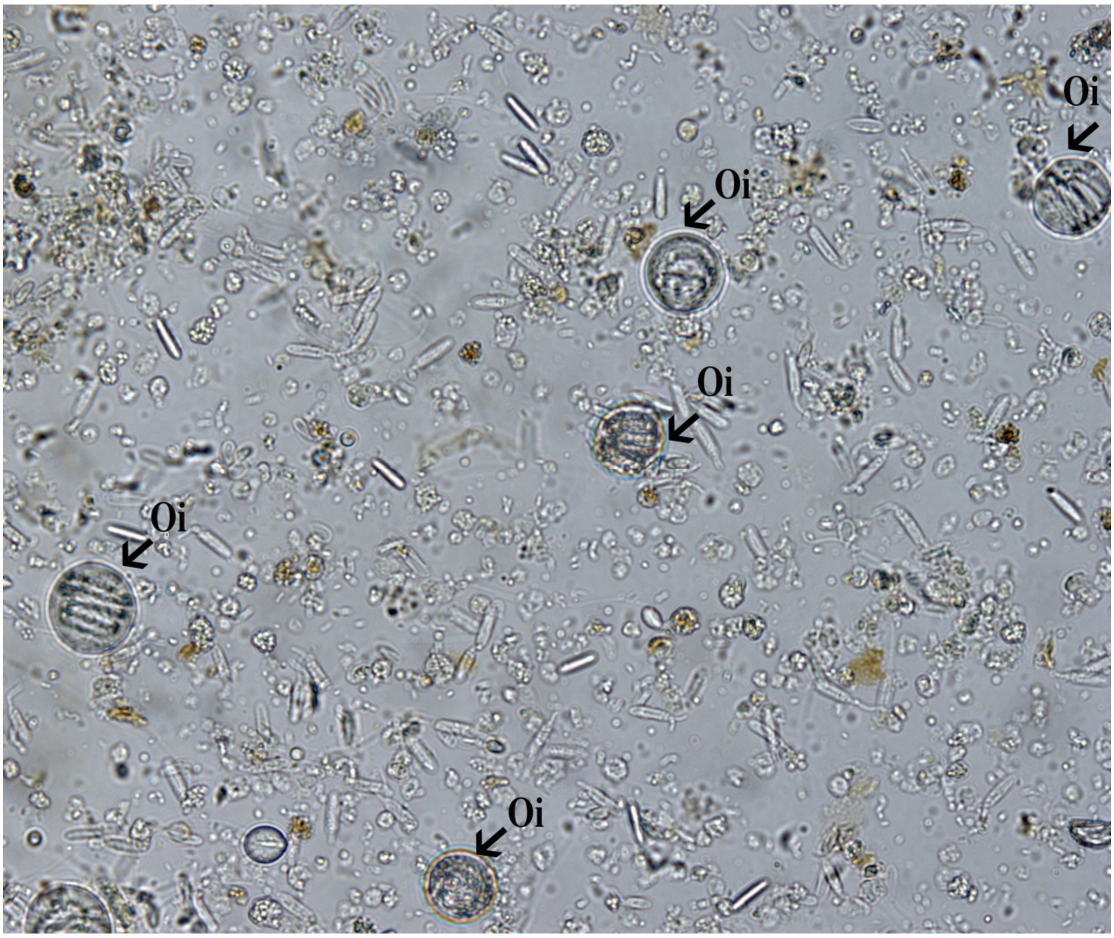

Figure S1A. Microscopic photography of nematocysts from the jellyfish *N. nomurai* individual J1 collected in station K1 (magnification 40x). The picture was taken using a Zeiss microscope (Carl Zeiss, Oberkochen, Germany). Oi, O-isorhizas.

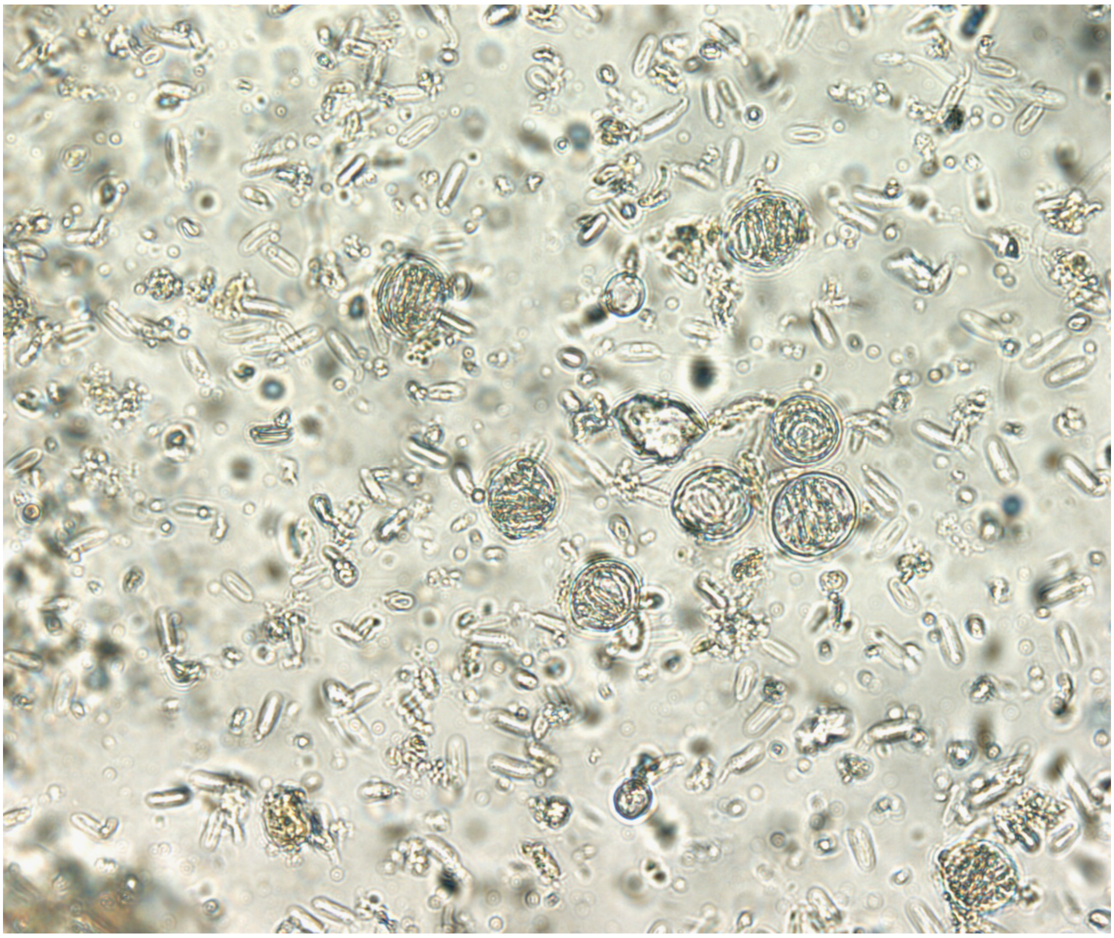

Figure S1B. Microscopic photography of nematocysts from the jellyfish *N. nomurai* individual J2 collected in station CJ-05 (magnification 40x). The picture was taken using a Zeiss microscope (Carl Zeiss, Oberkochen, Germany).

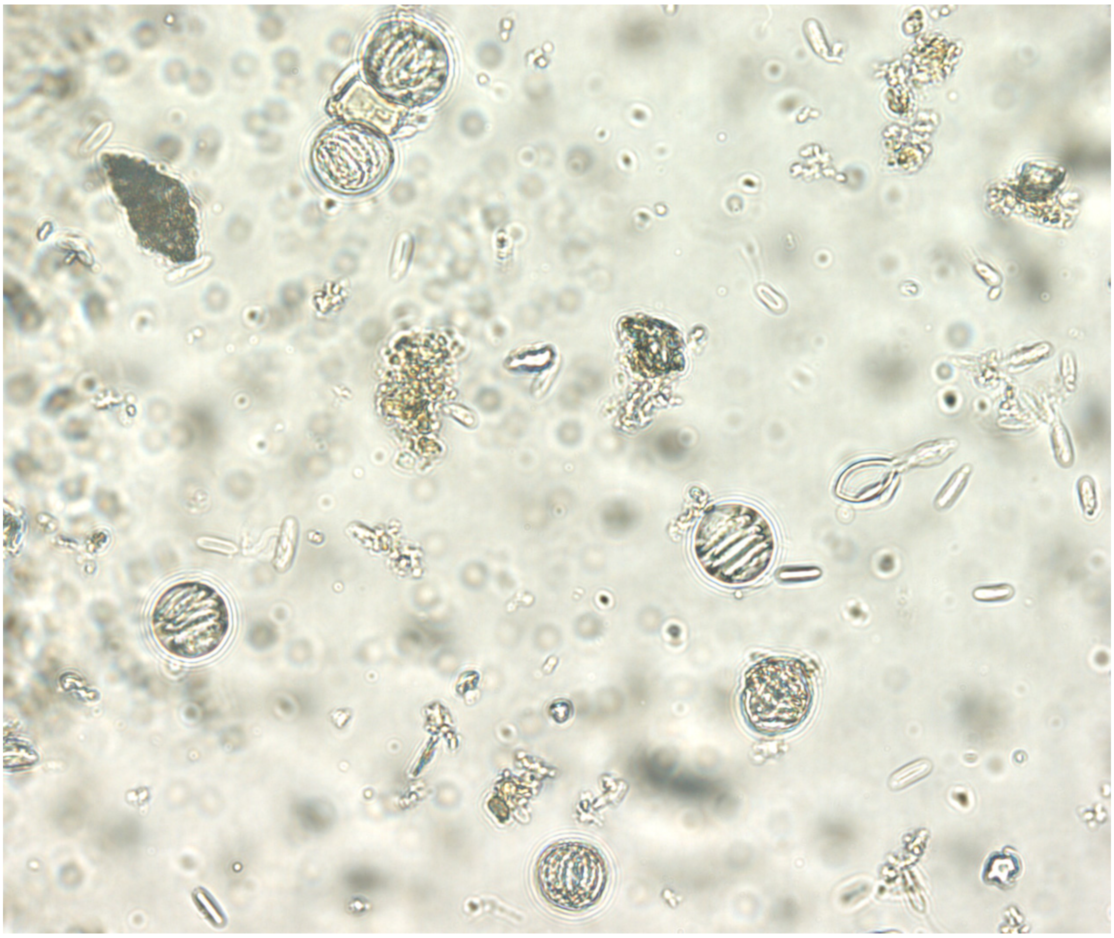

Figure S1C. Microscopic photography of nematocysts from jellyfish *N. nomurai* individual J3 collected in station 3300-02 (magnification 40x). The picture was taken using a Zeiss microscope (Carl Zeiss, Oberkochen, Germany).

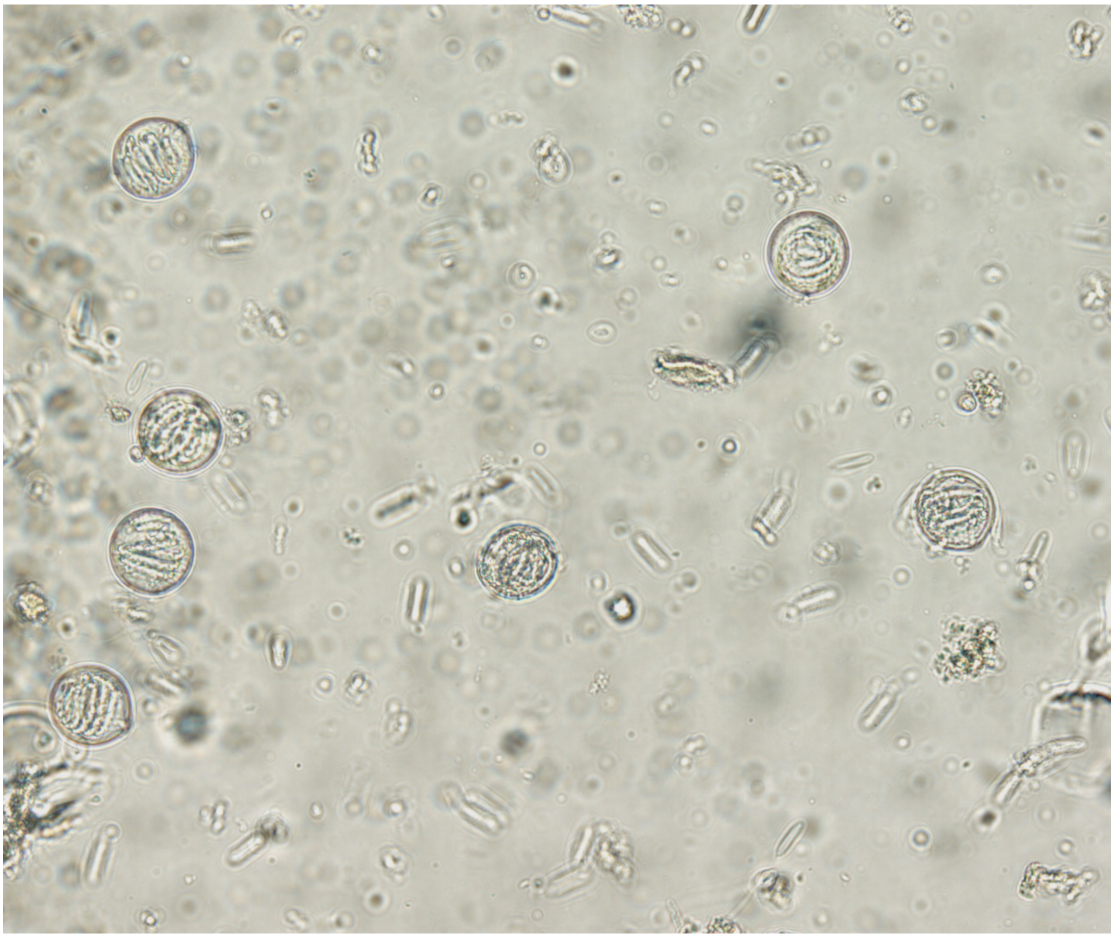

Figure S1D. Microscopic photography of nematocysts from the jellyfish *N. nomurai* individual J4 collected in station 3400-06 (magnification 40x). The picture was taken using a Zeiss microscope (Carl Zeiss, Oberkochen, Germany).

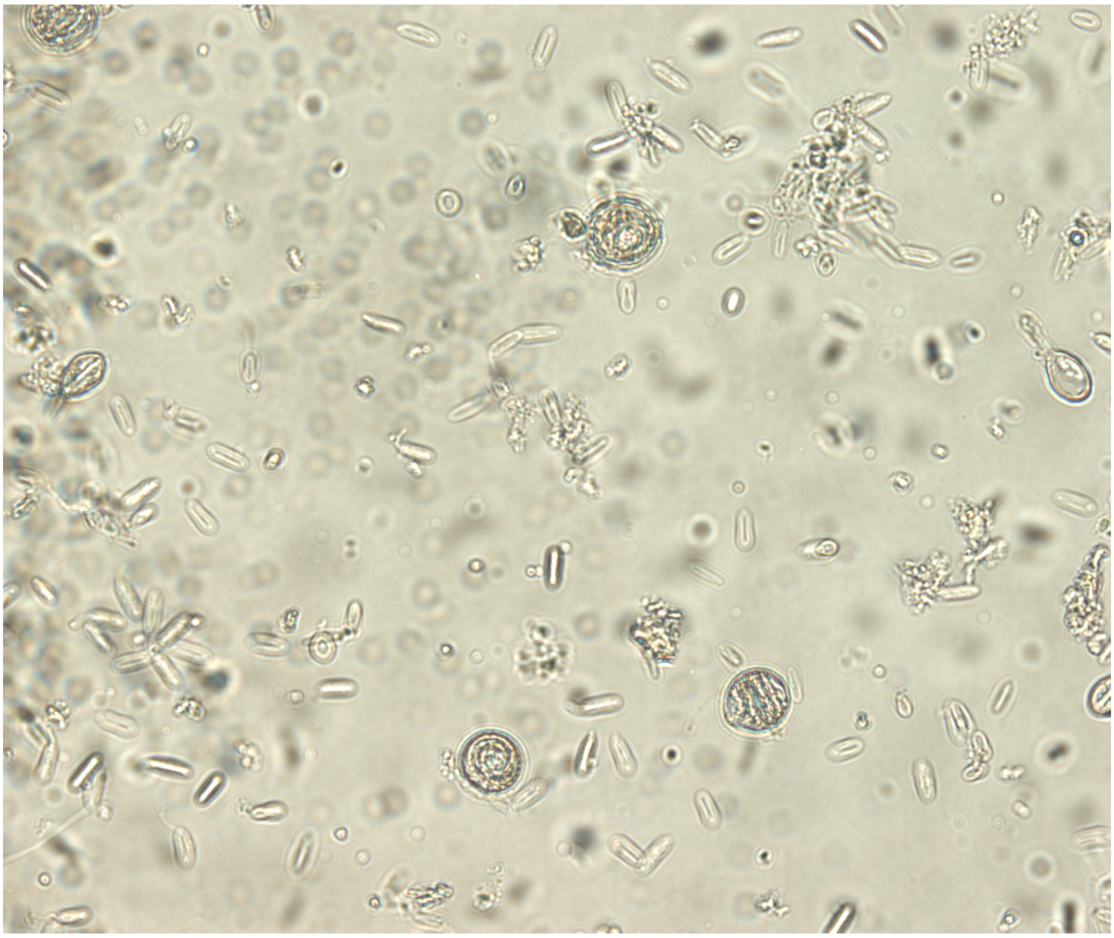

Figure S1E. Microscopic photography of nematocysts from the jellyfish *N. nomurai* J5 collected in station 3500-06 (magnification 40x). The picture was taken using a Zeiss microscope (Carl Zeiss, Oberkochen, Germany).

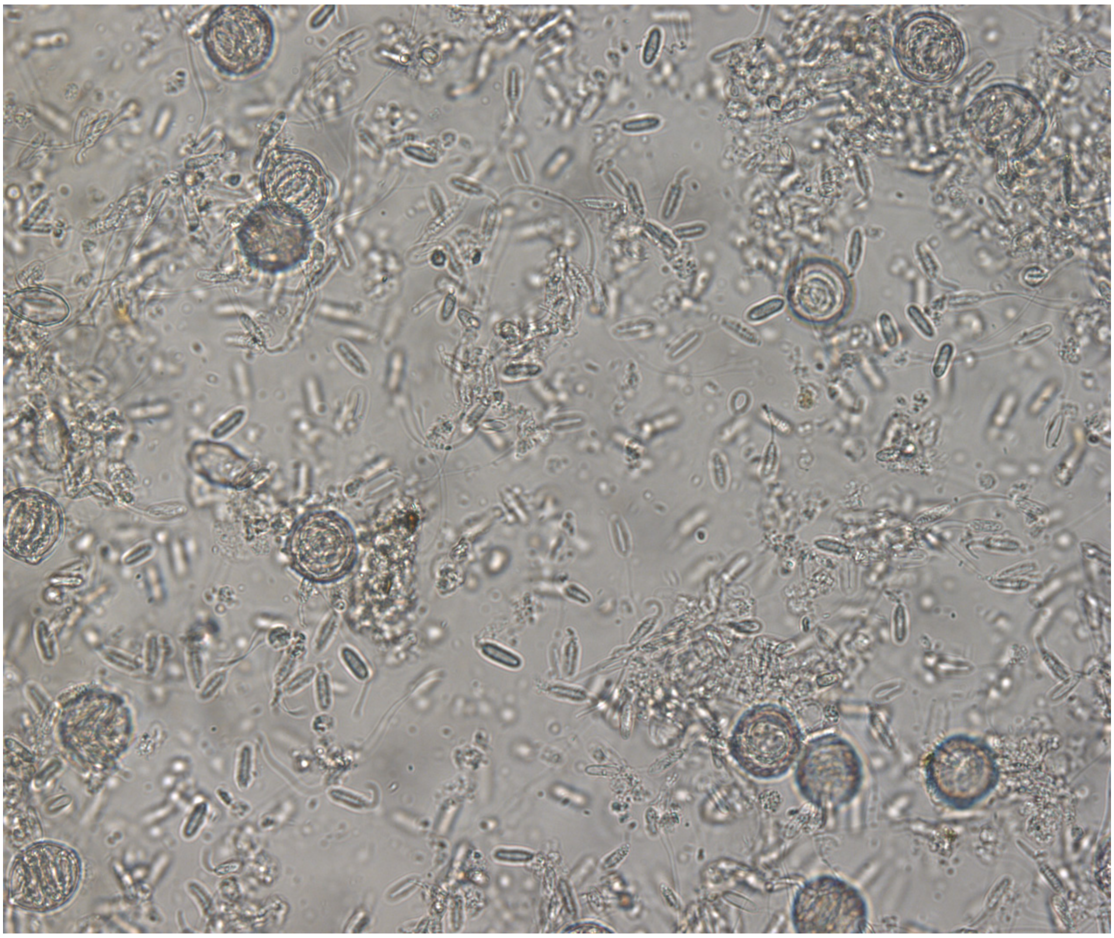

Figure S1F. Microscopic photography of nematocysts from the jellyfish *N. nomurai* individual J6 collected in station 3500-05 (magnification 40x). The picture was taken using a Zeiss microscope (Carl Zeiss, Oberkochen, Germany).

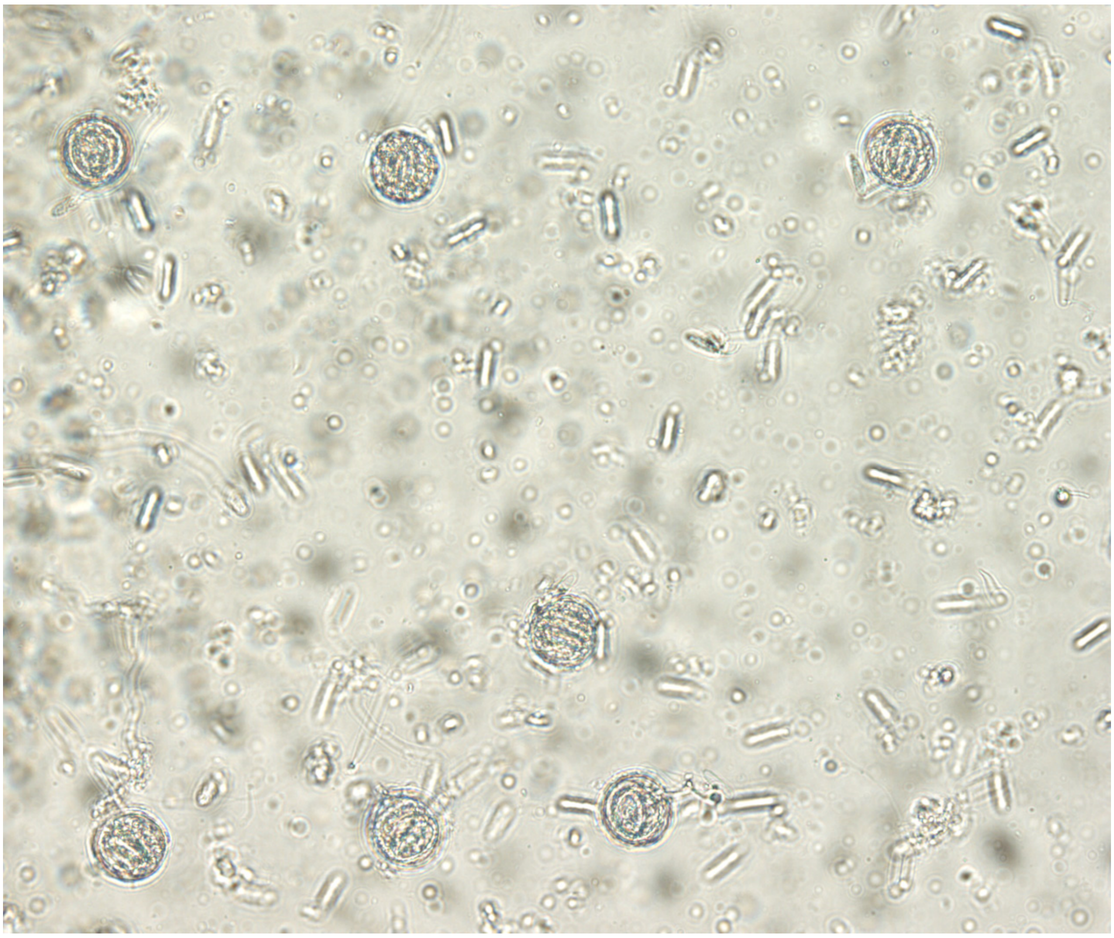

Figure S1G. Microscopic photography of nematocysts from the jellyfish *N. nomurai* individual J7 collected in station 3600-07 (magnification 40x). The picture was taken using a Zeiss microscope (Carl Zeiss, Oberkochen, Germany).

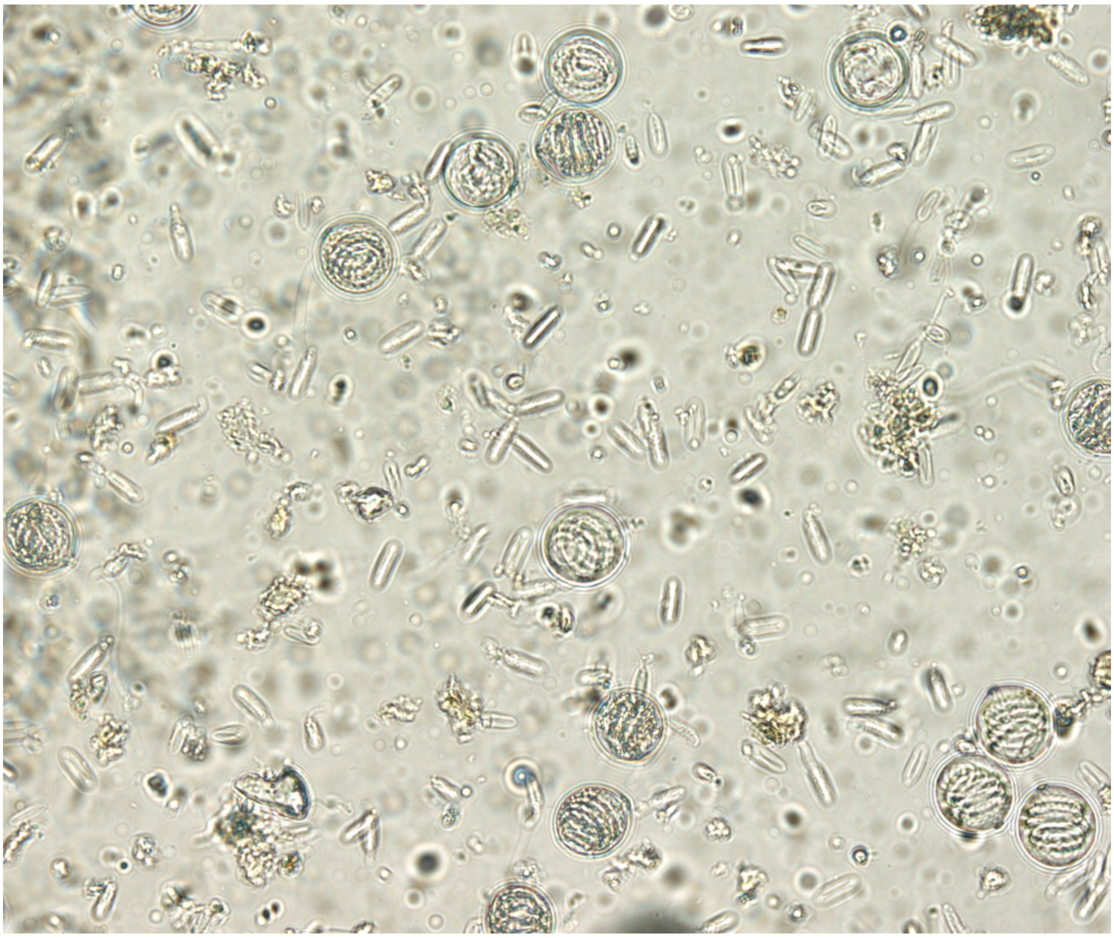

Figure S1H. Microscopic photography of nematocysts from the jellyfish *N. nomurai* individual J8 collected in station 3700-01 (magnification 40x). The picture was taken using a Zeiss microscope (Carl Zeiss, Oberkochen, Germany).

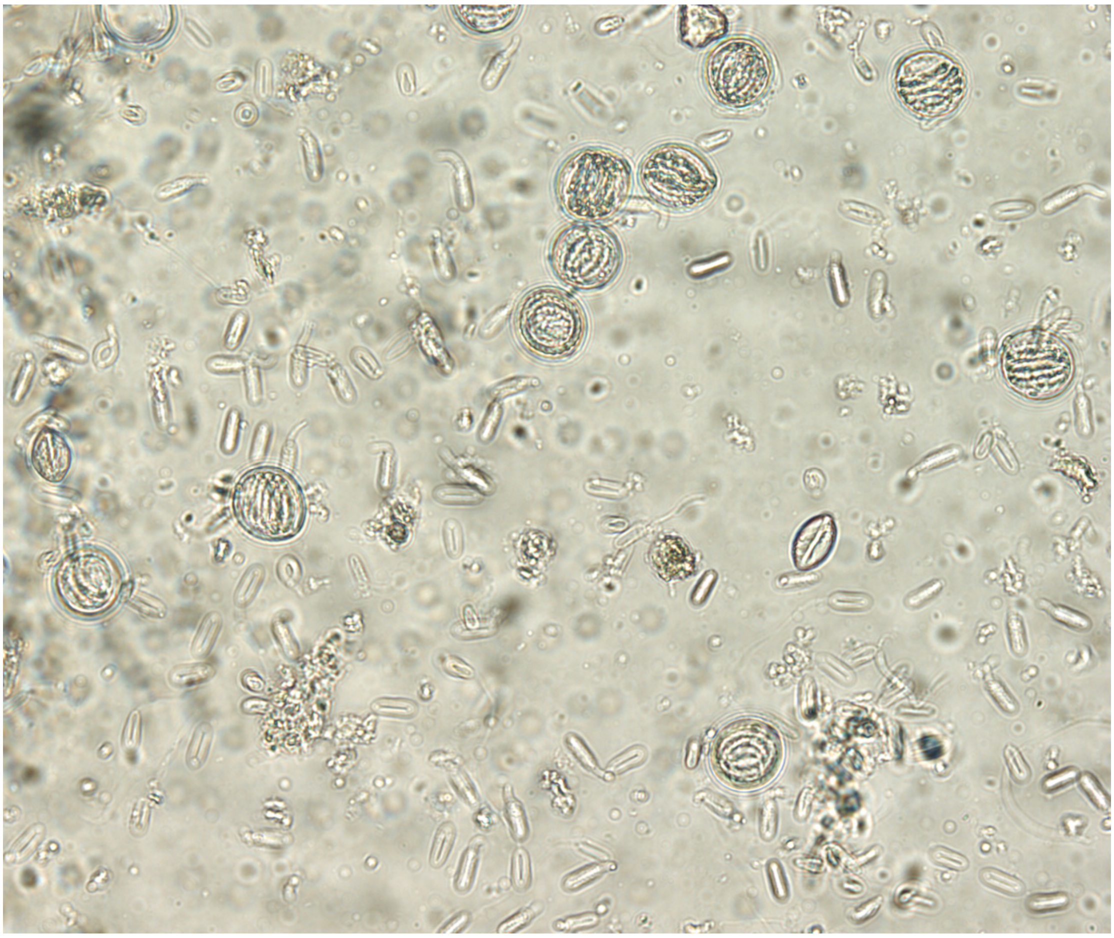

Figure S1I. Microscopic photography of nematocysts from the jellyfish *N. nomurai* individual J9 collected in station 3700-03 (magnification 40x). The picture was taken using a Zeiss microscope (Carl Zeiss, Oberkochen, Germany).

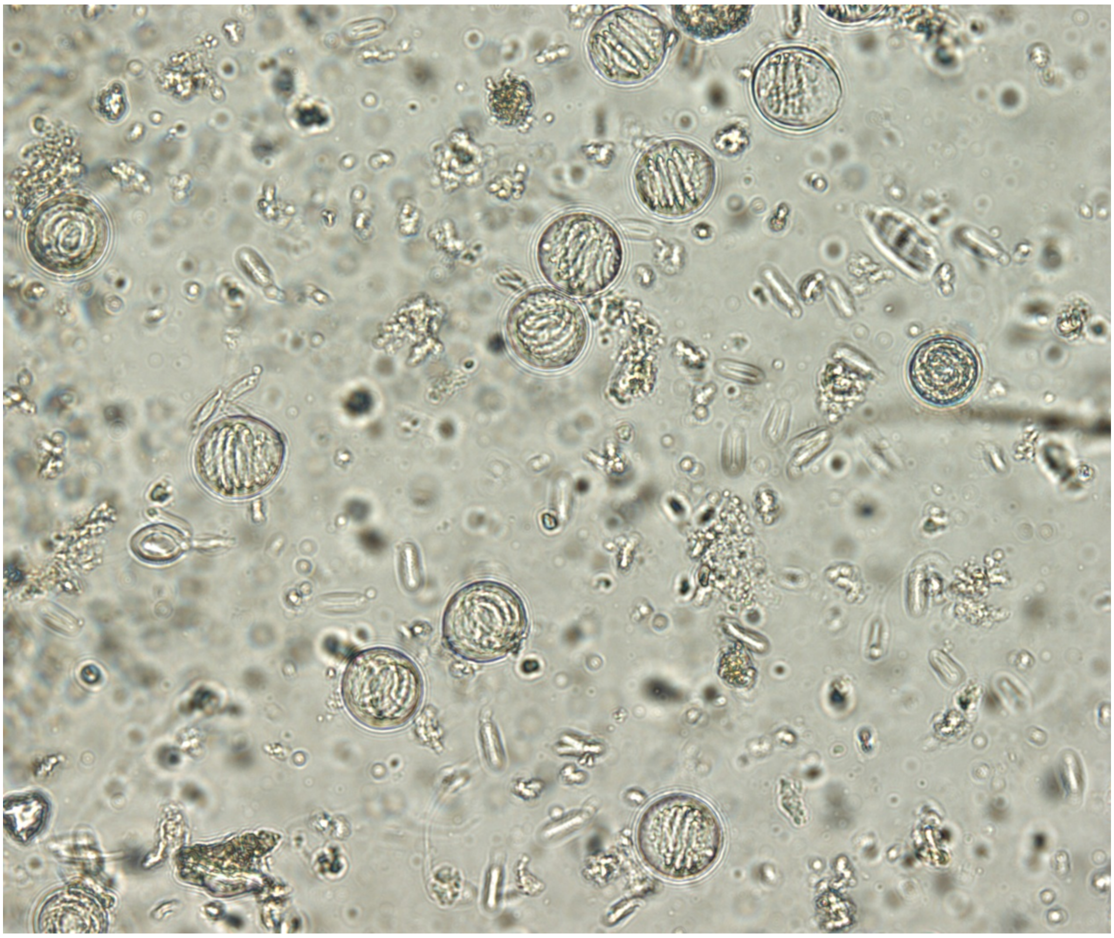

Figure S1J. Microscopic photography of nematocysts from the jellyfish *N. nomurai* individual J10 collected in station B-06 (magnification 40x). The picture was taken using a Zeiss microscope (Carl Zeiss, Oberkochen, Germany).

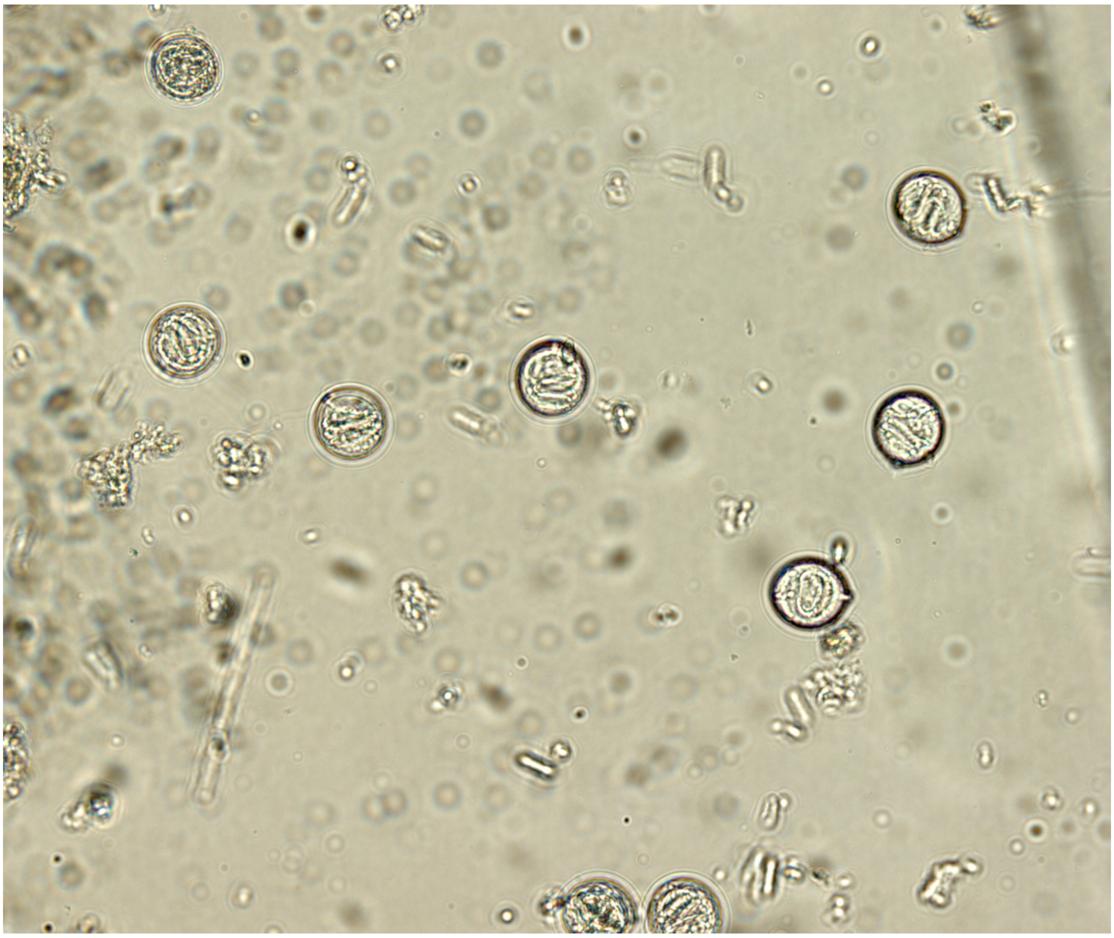

Figure S1K. Microscopic photography of nematocysts from the jellyfish *N. nomurai* individual J11 collected in station 3875-02 (magnification 40x). The picture was taken using a Zeiss microscope (Carl Zeiss, Oberkochen, Germany).

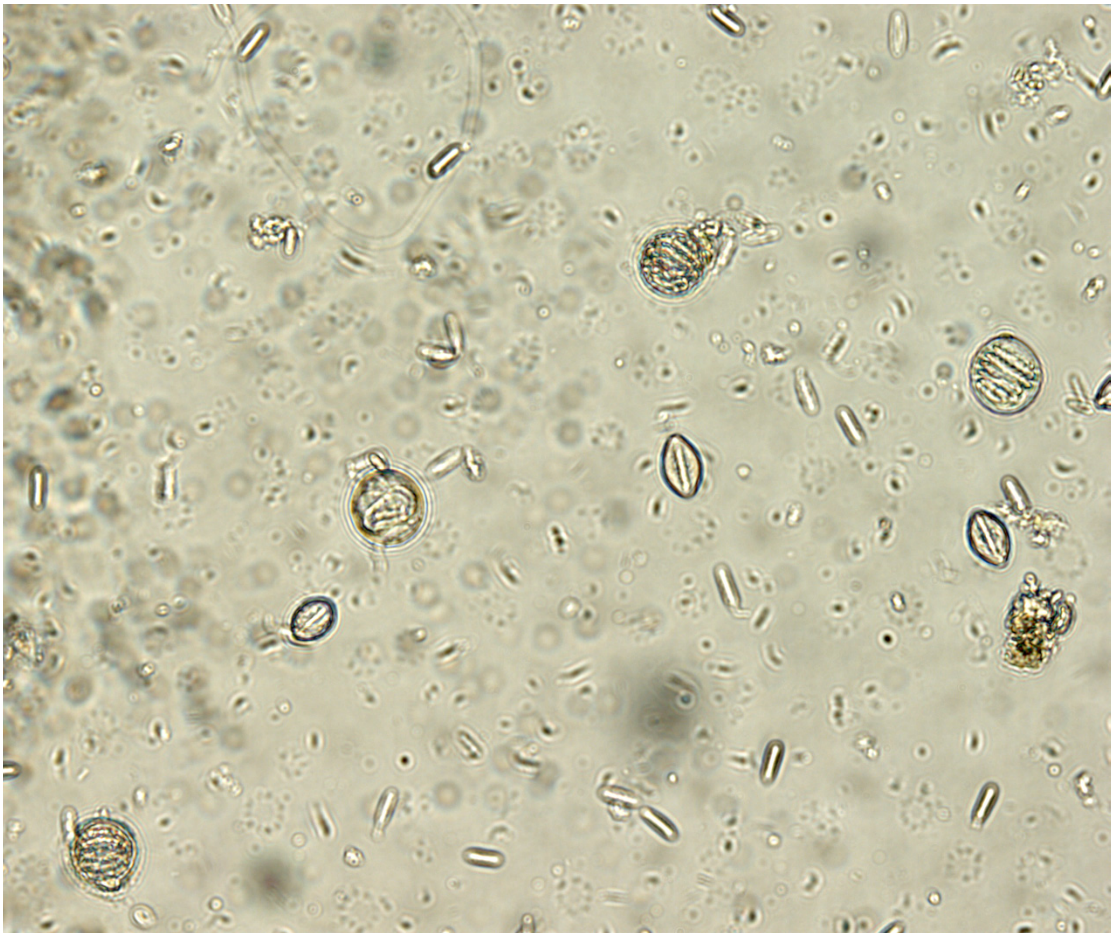

Figure S1L. Microscopic photography of nematocysts from the jellyfish *N. nomurai* individual J12 collected in station 3875-05 (magnification 40x). The picture was taken using a Zeiss microscope (Carl Zeiss, Oberkochen, Germany).

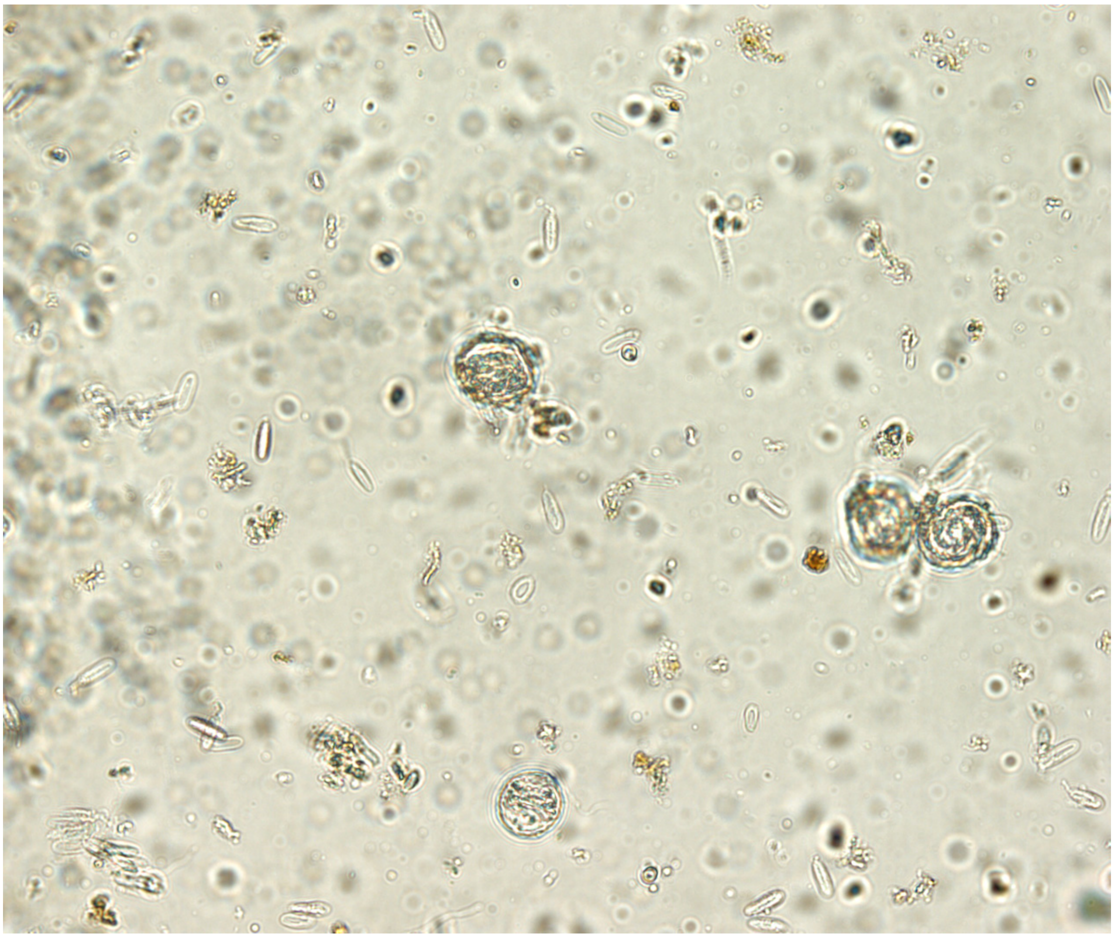

Figure S1M. Microscopic photography of nematocysts from the jellyfish *N. nomurai* individual J13 collected in station 3500-06 (magnification 40x). The picture was taken using a Zeiss microscope (Carl Zeiss, Oberkochen, Germany).

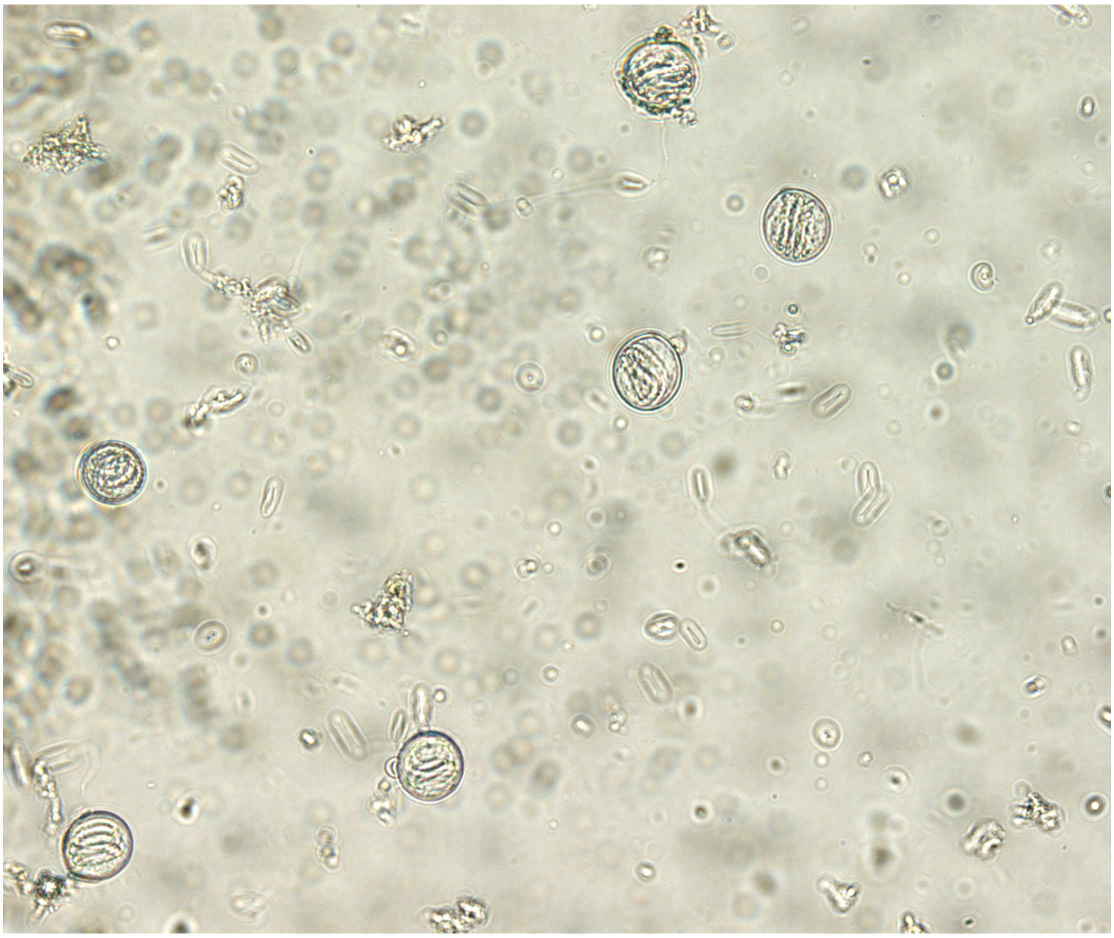

Figure S1N. Microscopic photography of nematocysts from the jellyfish *N. nomurai* individual J14 collected in station 3400-06 (magnification 40x). The picture was taken using a Zeiss microscope (Carl Zeiss, Oberkochen, Germany).

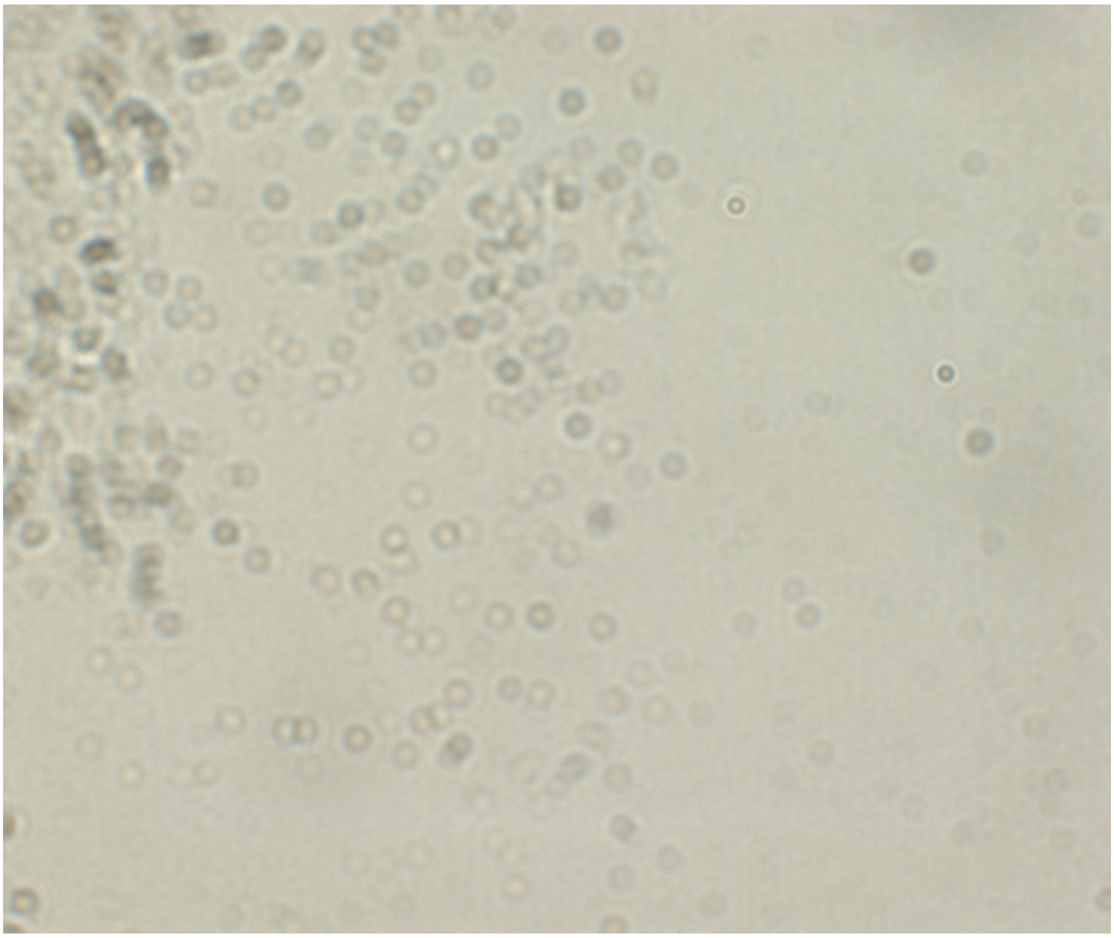

Figure S1O. Microscopic photography of blank control (20 mM PBS, pH 7.4) without nematocysts (magnification 40x). The objective lens is contaminated by unidentified subject. The picture was taken using a Zeiss microscope (Carl Zeiss, Oberkochen, Germany).

Zymography of hyaluronidases

Hyaluronidases activity of jellyfish venom were examined by zymography as described in our previous study[1]. Briefly, the electrophoresis gel containing substrate was prepared by adding the substrate hyaluronic acid (0.17 mg/mL, final concentration) into the 12% SDS-PAGE resolving gel. Then, about 10 μg of venom proteins from jellyfish individuals J1-J14 were loaded and run at 120V and 4°C for 120 min under non-reducing conditions. To maintain the hyaluronidase activity as much as possible, electrophoresis apparatus was surrounded by ice. After

electrophoresis, the gels were washed twice with 2.5% Triton X-100 for 40 min, and then incubated twice for another 30 min in the assay buffer (100 mM sodium acetate buffer containing 150 mM NaCl, pH 6.0) to remove the Triton X-100. After incubation for 24h at 37°C in the assay buffer, the gel was then stained with 0.1% alcian blue for 3 h and destained in 5% acetic acid until clear translucent bands appeared against the dark blue background. The results were displayed in Fig. S2.

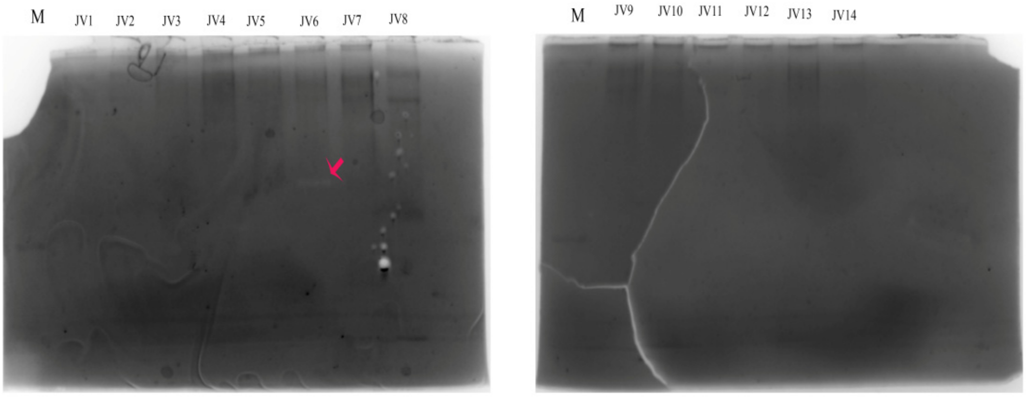

Figure S2. Zymogram of hyaluronidase. The substrate hyaluronic acid (0.17 mg/ml) was incorporated into the resolving gel for electrophoresis together with 10 μg of JV1-JV14 under non-reducing conditions. The substrate gel was stained with 0.1% alcian blue and the presence of clear translucent bands against the dark blue background reveals the existence of hyaluronidase. The arrows indicate the existence of hyaluronidase in JV6. M, markers.

Reference

1 Yue, Y. *et al.* Functional Elucidation of *Nemopilema nomurai* and *Cyanea nozakii* Nematocyst Venoms' Lytic Activity Using Mass Spectrometry and Zymography. *Toxins* **9**, 47 (2017).
